# Spectral fingerprints or spectral tilt? Evidence for distinct oscillatory signatures of memory formation

**DOI:** 10.1371/journal.pbio.3000403

**Published:** 2019-07-29

**Authors:** Marie-Christin Fellner, Stephanie Gollwitzer, Stefan Rampp, Gernot Kreiselmeyr, Daniel Bush, Beate Diehl, Nikolai Axmacher, Hajo Hamer, Simon Hanslmayr

**Affiliations:** 1 Department of Neuropsychology, Institute of Cognitive Neuroscience, Ruhr University Bochum, Bochum, Germany; 2 Epilepsy Center, Department of Neurology, University Hospital Erlangen, Erlangen, Germany; 3 Department of Neurosurgery, University Hospital Erlangen, Erlangen, Germany; 4 Institute of Cognitive Neuroscience, University College London, London, United Kingdom; 5 Department of Clinical Neurophysiology, National Hospital for Neurology and Neurosurgery, London, United Kingdom; 6 Department of Clinical and Experimental Epilepsy, Institute of Neurology, University College London, London, United Kingdom; 7 School of Psychology, Centre for Human Brain Health, University of Birmingham, Birmingham, United Kingdom; University of California - Berkeley, UNITED STATES

## Abstract

Decreases in low-frequency power (2–30 Hz) alongside high-frequency power increases (>40 Hz) have been demonstrated to predict successful memory formation. Parsimoniously, this change in the frequency spectrum can be explained by one factor, a change in the tilt of the power spectrum (from steep to flat) indicating engaged brain regions. A competing view is that the change in the power spectrum contains several distinct brain oscillatory fingerprints, each serving different computations. Here, we contrast these two theories in a parallel magnetoencephalography (MEG)–intracranial electroencephalography (iEEG) study in which healthy participants and epilepsy patients, respectively, studied either familiar verbal material or unfamiliar faces. We investigated whether modulations in specific frequency bands can be dissociated in time and space and by experimental manipulation. Both MEG and iEEG data show that decreases in alpha/beta power specifically predicted the encoding of words but not faces, whereas increases in gamma power and decreases in theta power predicted memory formation irrespective of material. Critically, these different oscillatory signatures of memory encoding were evident in different brain regions. Moreover, high-frequency gamma power increases occurred significantly earlier compared to low-frequency theta power decreases. These results show that simple “spectral tilt” cannot explain common oscillatory changes and demonstrate that brain oscillations in different frequency bands serve different functions for memory encoding.

## Abstract 2

There are two competing explanations for electrophysiological signatures during cognitive processes. One assumes simultaneous increases in high frequencies paired with decreases in low frequencies, whereas the other suggests that different frequencies index separate oscillatory processes. This study reports data that support the latter view.

## Introduction

Understanding the neural processes that mediate encoding of new memories is fundamental. Encoding processes are at the first stage of transforming transient experiences into memories, which essentially make us who we are. The subsequent memory paradigm allows investigating these processes by contrasting neural activity during later-remembered events with activity during later-forgotten events at encoding [1]. Electrophysiological methods, like intracranially recorded electroencephalography (EEG) or EEG/magnetoencephalography (MEG), are particularly promising to offer a mechanistic understanding of memory formation processes. M/EEG and intracranial EEG (iEEG) index neural synchronization and desynchronization processes [2,3], which have been directly linked to synaptic plasticity [4–6]. A number of subsequent memory studies demonstrate that memory formation is indicated not by one particular frequency but instead by complex changes in multiple frequencies ranging from 2 to 100 Hz, encompassing theta, alpha, beta, and gamma frequency bands [7,8]. A common finding is that power decreases in low frequencies (<30 Hz) paired with increases in high-frequency power (>40 Hz) are beneficial for memory formation. This change in the spectral pattern can result from two different processes: (1) a change in the “spectral tilt” (i.e., a shift from low-frequency activity to high-frequency activity [9–11]) or (2) changes at multiple distinct frequency bands related to distinct subprocesses involved in memory formation. Here, we contrast these two frameworks in a subsequent memory paradigm and show that memory-related frequency components can be dissociated on three levels: experimental condition, temporal dynamics, and brain regions.

Decreases in low-frequency power are often accompanied by increases in high-frequency power during various tasks. This is especially true for alpha/beta power decreases and gamma power increases [12–14]. Human EEG shows a 1/f-like characteristic whereby power decreases with increasing frequency [11,15]. A change in the “tilt” of this 1/f spectrum can parsimoniously explain such low-frequency decreases accompanied by gamma power increases [8,11,16]. The “spectral tilt” idea makes no assumptions about synchronization processes but instead views a shift from low- to high-frequency activity as a proxy for increased neural firing [10,17,18]. Evidence for the spectral tilt theory comes from recent brain-stimulation studies that were able to boost memory encoding by stimulating specifically during periods characterized by “bad memory states,” i.e., increased low- and decreased high-frequency activity [19,20].

The spectral tilt framework makes three specific predictions, which will be tested here: (1) Increases in gamma power should be accompanied by decreases in alpha/beta and theta power and vice versa. Therefore, it should not be possible to experimentally dissociate gamma power increases from alpha/beta and theta power decreases. (2) Decreases in low-frequency (alpha/beta/theta) power and increases in high-frequency (gamma) power should occur in strongly overlapping brain regions; and (3) low-frequency decreases and high-frequency increases should occur at the same time. In sum, the “tilt” assumption suggests that high-frequency increases and low-frequency decreases reflect the same process. Following this assumption, many iEEG studies confine analysis to broadband high-frequency power changes, presuming that high-frequency increases reliably map brain activity [21–25]

The “spectral fingerprints” framework assumes that oscillatory changes in different frequencies indicate different neural processes in different brain regions [26], each reflecting a specific function in the service of memory [7,27,28]. The framework of “spectral fingerprints” has a specific relevance when studying memory formation, because the idea that several distinct subprocesses contribute to memory formation is integral to many memory models [29–31]. Prior work has linked different oscillatory changes to assumed memory subprocesses. For instance, theta oscillations have been related to binding processes in a medial temporal lobe (MTL) network [32–34], whereas alpha/beta power decreases have been related to semantic processing during memory encoding [35–37]. Gamma power increases in sensory areas have been suggested to reflect locally synchronized activity and to indicate bottom-up sensory processing [3,38]. The “spectral fingerprints” framework therefore arrives at very different predictions compared to the spectral “frequency tilt” framework. Specifically, it suggests that it should be possible to experimentally dissociate gamma power increases from theta and alpha/beta power decreases. It further suggests that the power changes of different frequency bands could occur in different brain regions and at different time points.

**Fig 1:**
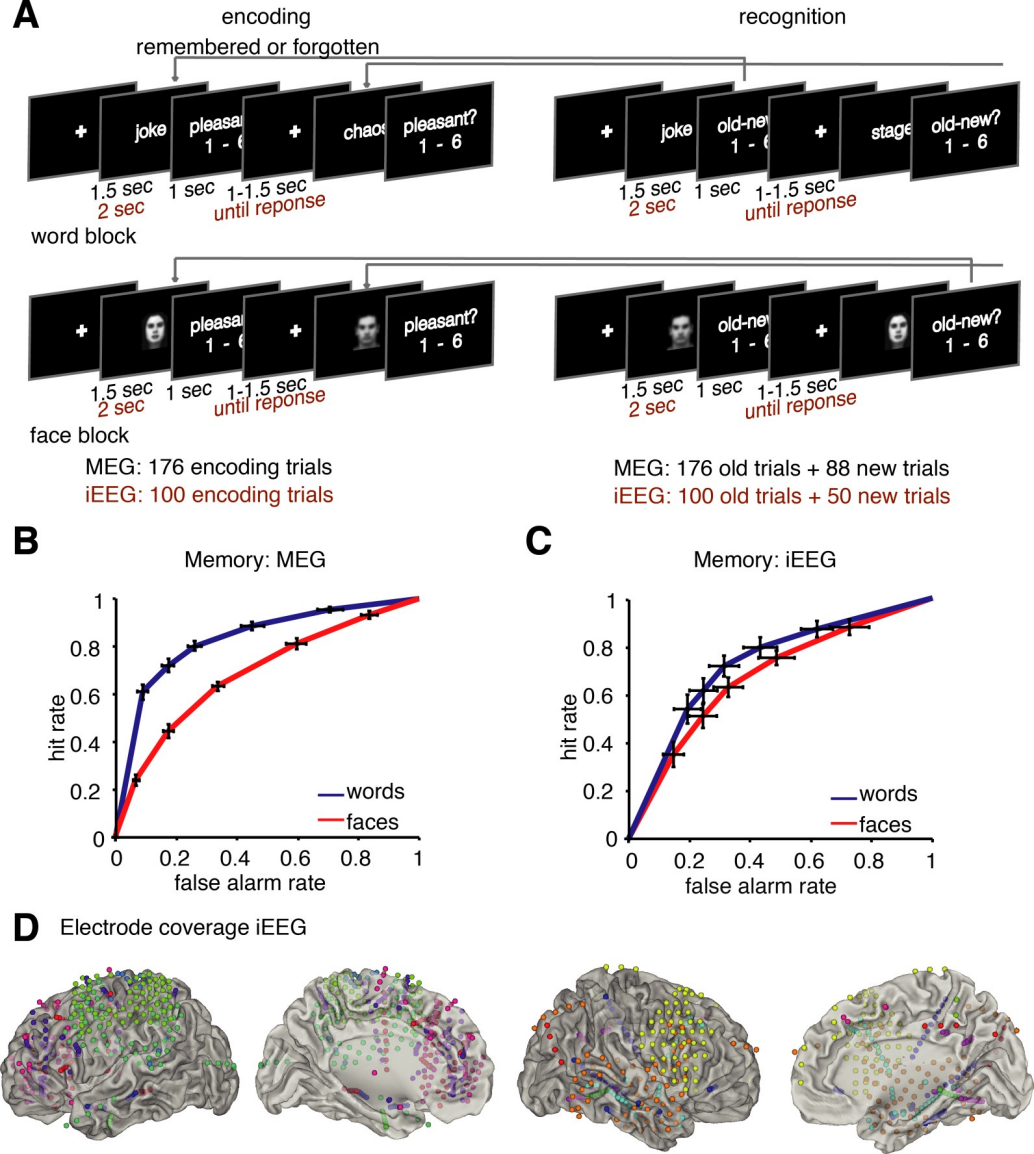
(A) Paradigm. The experiment was split in two blocks: one block for word encoding and recognition and one for faces, respectively. The paradigm was slightly adapted for the iEEG patient sample. Behavioral performance in the MEG dataset (B) and iEEG dataset (C). ROC curves show memory performance for faces and words; error bars plot SEM for each rating. A left upward shift of the ROC indicates higher recognition performance—i.e., more hits and fewer false alarms. (D) Electrode coverage in the iEEG patient sample: The bipolar referenced virtual electrode locations of each contact included in the reported analysis are plotted here; colors code different patients. Data and scripts underlying this figure are deposited here: https://osf.io/3csku/. iEEG, intracranial electroencephalography; MEG, magnetoencephalography; ROC, receiver operating characteristic.

To test the two frameworks against each other, we employed the same memory task while recording iEEG in patients and MEG in healthy participants. It is important to note that many subsequent memory studies arguing in favor of spectral fingerprints used noninvasive MEG/EEG [32,37], whereas most studies finding evidence for a spectral tilt used intracranially recorded EEG [8,20]. To investigate whether specific spectral fingerprints can be dissociated by varying encoding demands, we utilized the well-established finding that memory encoding of words is crucially different from encoding of unfamiliar faces. Specifically, encoding of words heavily depends on semantic processing [39], whereas encoding of unfamiliar faces solely depends on visual processing [40,41]. Therefore, we recorded MEG and iEEG during encoding of words and faces (see Fig 1A) to rule out differences in recording methods as a confounding factor (i.e., higher sensitivity of iEEG to capture high-frequency dynamics [42,43]). This combined measurement also allowed us to capture changes in a large frequency range (2–100 Hz) on a whole-brain and at a more local level.

## Results

### Behavioral results

Memory performance is shown by means of receiver operating characteristic (ROC) curves in Fig 1B and 1C, depicting memory performance in the MEG sample (healthy participants) and the iEEG sample (patients). Recognition performance in the MEG sample was higher for words (mean d′ = 1.99) than for faces (mean d′ = 0.83, t_19_ = 7.836, *p* < 0.0001). A similar effect was observed in the iEEG sample, which was slightly weaker likely because of the higher variance in performance between patients (mean d′ words = 1.37 versus mean d′ faces = 0.90; t_12_ = 2.12, *p* = 0.052). A follow-up analysis directly comparing memory performance in the MEG and iEEG samples revealed better memory performance in the MEG compared to the iEEG sample (ANOVA material × group, difference d′ MEG versus iEEG, F_1,31_ = 41.18, *p* < 0.0001). The difference in memory performance between words and faces was also more pronounced in the MEG compared to the iEEG sample (interaction material × group, F_1,31_ = 7.40, *p* = 0.011). These results are in line with previous studies demonstrating the difficulty of memorizing unfamiliar faces [41,44]. The difference in recognition performance already hints at different processes involved in encoding of words in contrast to faces.

### Material-specific effects: Lower-frequency bands

The experiment involved two main factors: material (words versus faces) and subsequent memory (remembered versus forgotten). In a first step, we analyzed overall differences of material, i.e., differences in power between processing a word or face item independent of memory. This allowed us to identify time-frequency windows of interest for later analysis and served as a first test to check for colocalized low- and high-frequency power decreases/increases.

**Fig 2:**
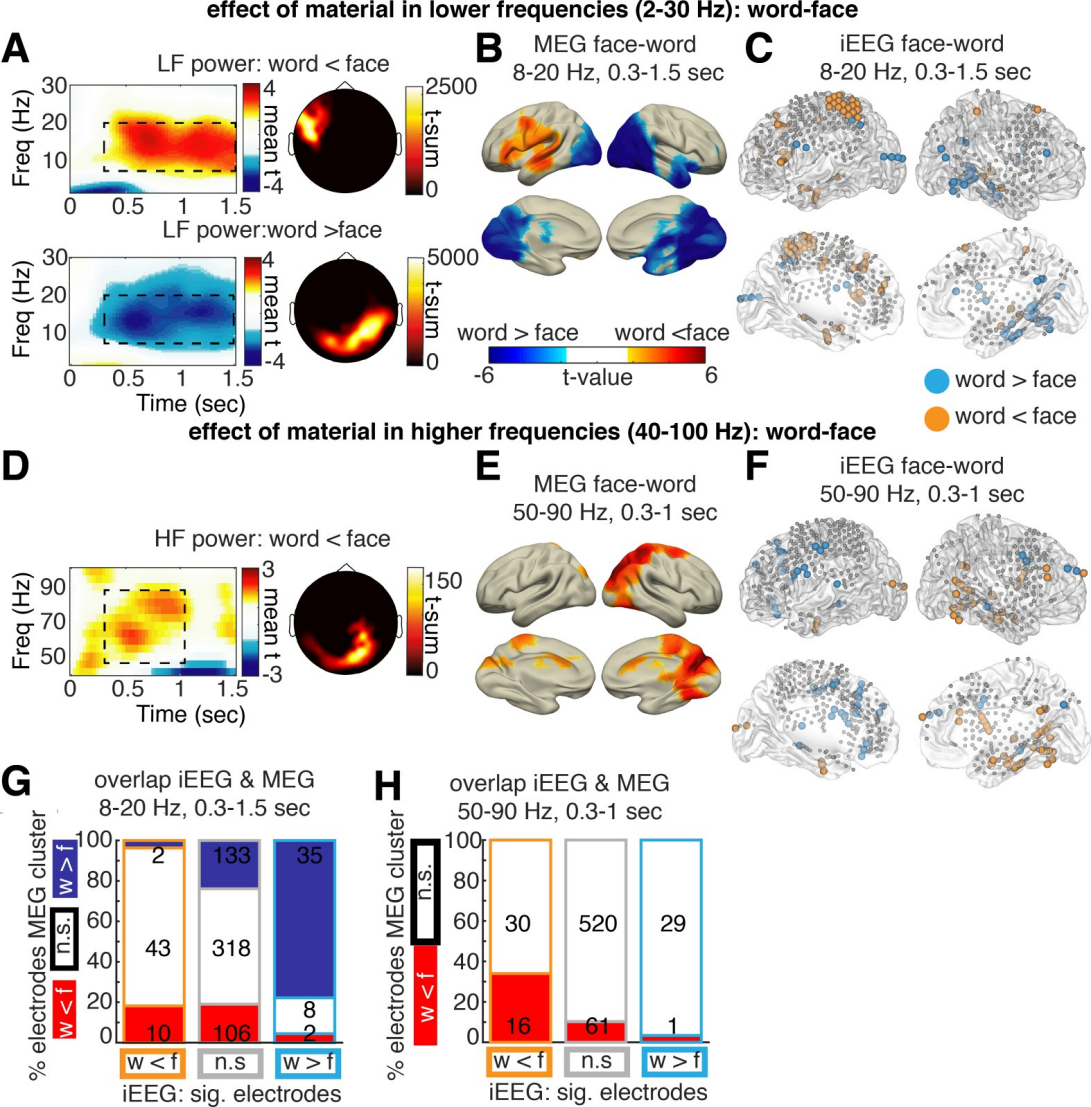
MEG main effect of material: Word-faces. (A) Main effect of material: word versus face condition irrespective of memory: significant clusters (*p*_corr_ < 0.05) returned by a cluster permutation statistic clustering across sensors, frequencies (“Freq”), and channels. One cluster showed a greater decrease in alpha/beta power for words relative to faces at left frontal sensors; another cluster showed a stronger decrease in alpha/beta power for faces relative to words at posterior sensors. Time-frequency plots show average t-values in the significant sensor cluster highlighted in the topography plot. (B) Significant sources (*p*_corr_ < 0.05) showing a main effect of material in alpha/beta power in the time-frequency window identified in sensor-level data. (C) Electrodes (*p* < 0.05, uncorrected) in iEEG showing a significant change in alpha/beta power in the respective time-frequency window identified in MEG. (G) The overlap of MEG and iEEG material effects is visualized by plotting for each category of iEEG electrodes (“w < f”: light blue, “w > f”: orange, or no significant difference [“n.s.”]: gray), the percentage of electrodes located in areas of significant (“sig.”) MEG source clusters (clusters of w < f: red, w > f: blue, or no significant difference: white). The numbers plotted on top of the bars denote the absolute numbers of electrodes in each source area respectively. (D-F and H) Corresponding analysis in the HF range. No significant MEG cluster emerged for word > face. Data and scripts underlying this figure are deposited here: https://osf.io/3csku/. HF, high frequency; iEEG, intracranial electroencephalography; LF, low frequency; MEG, magnetoencephalography.

Sensor-level analysis of the MEG data revealed significant differences in the lower frequencies, i.e., the alpha/beta band. A cluster permutation statistic across sensors, frequency bands (2–30 Hz), and time (0–1.5 s post stimulus) revealed two significant clusters spanning alpha/beta frequency bands (word < face, *p*_corr_ = .007, word > face, *p*_corr_ = .002; see Fig 2A and see S1 Fig for additional t-sum time-frequency plot): One cluster exhibited relative greater alpha/beta power decreases (8–20 Hz) for words than for faces, located at left frontal sensor sites at 0.3–1.5 s post stimulus. A second cluster exhibited relatively stronger alpha/beta power decreases (8–20 Hz) for faces than for words and was located at posterior sensors in the same time interval (0.3–1.5 s). In accordance with the sensor-level results, source analysis revealed significant clusters in regions commonly involved in word and face processing, respectively (Fig 2B): Stronger alpha/beta power decreases for words compared to faces (shown in red) were localized to areas typically involved in semantic processing encompassing the inferior and middle frontal gyrus, supramarginal gyrus, Heschl’s gyrus and temporal pole, and middle and superior temporal gyrus (peak t_19_ = −4.16 at MNI −54, 0, 50 corresponding to left precentral gyrus, word < face *p*_corr_ = 0.049, t-sum = −826.60). Stronger alpha/beta power decreases for faces compared to words (shown in blue) were localized to areas typically involved in visual processing (Fig 2B), spanning lingual, occipital middle, fusiform, temporal middle, and inferior gyrus in the right hemisphere. The peak of the source localization was in right inferior occipital gyrus (t_19_ = 9.04, MNI 46, 80, 10, source cluster: *p*_corr_ < 0.001, t-sum = 62,700.7). In summary, the material effects in MEG are in line with the hypothesis that areas involved in material-specific processing of stimuli show a relative decrease in alpha/beta power. A qualitatively similar picture of event-related alpha/beta increase in the range of 8–20 Hz also arises when plotting average stimulus-related changes to baseline power separately for faces and words (see S1 Fig for details). Decreases in alpha/beta power seem to closely map to areas involved in face and word processing as found with functional MRI (fMRI) [45].

In a next step, we assessed whether similar material-specific effects occurred in the iEEG data. To this end, the main effect of material in the time-frequency window identified in the MEG analysis (i.e., 8–20 Hz, 0.31.5 s) was calculated in each single electrode across the whole patient sample. Fig 2C shows all electrodes exhibiting a significant main effect of material (*p* < 0.05, uncorrected) in the alpha/beta frequency range, as assessed by a separate ANOVA with the factors material and memory in each electrode. Electrodes showing stronger alpha/beta power decreases for words relative to faces were spread across the left hemisphere (highlighted in orange in Fig 2C). In contrast, electrodes showing stronger alpha/beta power decreases for faces relative to words are predominantly located in the right posterior and ventral visual stream (highlighted in light blue in Fig 2C). The location of MEG sources and the distribution of significant iEEG electrodes for the words versus faces contrast showed a good degree of overlap visually (Fig 2A–2C). To formally test this overlap, a χ^2^ test of independence was calculated by counting the number of significant iEEG electrodes (Fig 2C) separately for areas inside and outside of significant source clusters in MEG source analysis (Fig 2B). The localization of significant iEEG electrodes and the MEG source clusters was not independent (Fig 2G, χ^2^_4_ = 79.79, *p* < 0.0001): iEEG electrodes were more likely to exhibit significant modulation of alpha/beta power (marked in light blue and orange) if located in regions that exhibit the same modulation in MEG (marked in blue and red). The results depicted in Fig 2G show that a face-selective electrode (uncorrected significant “w > f” alpha/beta difference, light blue) is more likely located in the “w > f” MEG cluster (dark blue). Furthermore, word-selective electrodes (uncorrected significant “w < f” alpha/beta difference, orange) are more likely located in the “w < f” MEG cluster (red) or in no MEG cluster than in the “w > f” MEG cluster (dark blue). This χ^2^ test requires categorization of electrodes into different groups, which we here did based on uncorrected *p* < 0.05. Two control analyses show that similar results are obtained when using a different categorization approach (i.e., direction of difference positive/negative) or when using a correlation analysis, which does not require any categorization at all (S2 Fig). Together, these findings demonstrate a good overlap of iEEG and MEG results, despite the different properties of iEEG and MEG in spatial resolution and spatial sampling.

### Material-specific effects: Higher-frequency bands

Material-specific effects in the higher-frequency range (40–100 Hz) were analyzed congruently with effects in the lower-frequency range. First, time-frequency windows of interest were identified via an open cluster permutation statistic across all MEG sensors, in a frequency range from 40 to 100 Hz and a time range from 0 to 1.5 s post stimulus. This analysis revealed three clusters exhibiting a significant increase in gamma power related to faces in contrast to words, all located at posterior sensors (approximately 50–90 Hz, 0.3–1.0 s post stimulus, *p*_corr_ = .011, *p*_corr_ = .011, *p*_corr_ = .046, Fig 2D, S1 Fig for additional t-sum time-frequency plot). These face-driven gamma power increases were localized to areas involved in visual processing (right cuneus, occipital superior gyrus, superior parietal gyrus and precuneus, peak in right cuneus, t_19_ = −8.07, MNI 16, −80, 30, source cluster *p* < 0.001, t-sum = −2,797.9). Plotting average power changes contrasted to prestimulus baseline show a qualitatively similar picture of event-related gamma increase in the range of 50–90 Hz stronger in the posterior “face” cluster than in “word” cluster (see S1 Fig for details). This result is consistent with the hypothesis that task active regions can be identified by increases in high-frequency power. However, this result was specific to face stimuli, as no clusters showing significant high-frequency power increases for word stimuli were identified.

Analogous to the low-frequency power analysis, the main effect of material in iEEG data was analyzed in the time-frequency window identified in the MEG data (50–90 Hz, 0.3–1 s). In Fig 2H, all electrodes showing significant increases in high-frequency (gamma) power for faces compared to words are highlighted in orange, and all electrodes showing gamma power increases for words are shown in blue (all *p* < 0.05, uncorrected). To quantify the overlap between the two modalities, a χ^2^ test of independence was calculated. Here, only electrodes with power increases for faces compared to words were tested, since no gamma power increases for words were found in the MEG data. The number of iEEG electrodes showing significant relative power increases for faces was higher in areas exhibiting the same effect in MEG (Fig 2H, χ^2^_2_ = 26.25, *p* < 0.0001; see S2 Fig for control analyses). Thus, changes in gamma power show a similar concordance of MEG and iEEG data as changes in alpha/beta power. Interestingly, in occipital areas, decreases in alpha/beta power seem to co-occur with increases in gamma power, whereas no such relationship was evident for areas related to the processing of words.

### Material-specific high-frequency effects in low-frequency clusters

**Fig 3:**
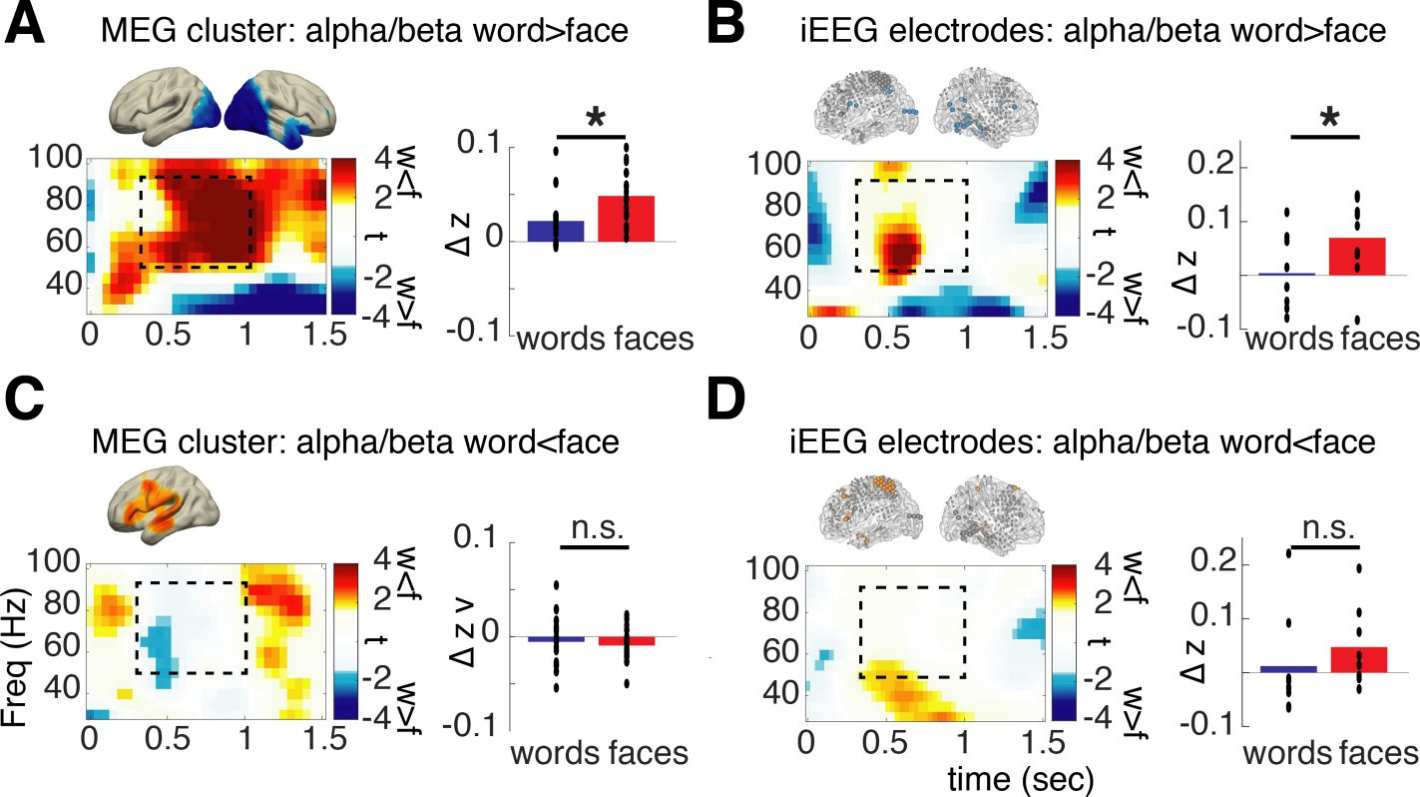
Material-specific high-frequency power changes in MEG source clusters and iEEG electrodes exhibiting material-specific power changes in the alpha/beta range. Time-frequency (“Freq”) plots show t-values for the contrast of words versus faces in MEG source clusters (A, C) and in iEEG electrodes (B, D). Bar plots show average power values for the word and face condition separately for the time-frequency window marked with dashed boxes; black dots show the respective single-subject averages. Asterisks mark significant differences depending on material (*p* < 0.05). Brain plots are replicated from Fig 2 to highlight the respective ROIs. Data and scripts underlying this figure are deposited here: https://osf.io/3csku/. iEEG, intracranial electroencephalography; MEG, magnetoencephalography; n.s., no significant difference; ROI, region of interest.

Material-specific effects were found in the form of low-frequency power decreases in the alpha/beta range and high-frequency gamma power increases. In line with the spectral tilt hypothesis, our results suggest that gamma power increases are related to decreases in alpha/beta power in occipital areas. However, no comparable increase in gamma power was evident in areas involved in word processing in the MEG using whole-brain statistics. To assure that such word-related high-frequency (gamma) power increases were not overlooked because of a lack of statistical power or weaker signal-to-noise ratio, we employed a region of interest (ROI) analysis focusing on gamma changes in MEG sources and iEEG electrodes exhibiting material-related alpha/beta power changes (Fig 3). High-frequency effects of material were assessed exclusively in MEG source clusters exhibiting material-related alpha/beta power changes (see Fig 2B for the respective clusters). In iEEG, an examination of gamma power changes was restricted to electrodes exhibiting material-related alpha/beta power changes. To calculate a random-effects group statistic in the iEEG data (that is comparable to the MEG data), power spectra were averaged in each patient across all electrodes exhibiting a significant negative or positive main effect of material in alpha/beta power. For both modalities, we then compared gamma power effects in those clusters (MEG) or electrodes (iEEG) exhibiting material-specific low-frequency power decreases.

In the clusters/electrodes that exhibited face-specific alpha/beta power decreases, a concurrent increase in gamma power was evident, which parallels the above findings. Specifically, both modalities, MEG and iEEG, showed a significantly stronger gamma power increase for faces compared to words (Fig 3A and 3B, MEG: t_19_ = −5.733, *p* < 0.0001, iEEG: t_8_ = −2.337, *p* = 0.0476). Concerning the clusters/electrodes exhibiting word-specific alpha/beta power decreases, however, no colocalized increases in high-frequency (gamma) power were evident (Fig 3C and 3D, MEG: t_19_ = 0.60, *p* = 0.56, iEEG: t_7_ = −1.260, *p* = 0.283). This pattern of results suggests that the co-occurrence of gamma increases and alpha/beta power decreases may be limited to specific brain areas. In posterior sensory-processing regions, our results agree with the spectral tilt hypothesis, showing increases in gamma power co-occurring with decreases in low-frequency power. However, in left lateralized areas exhibiting strong alpha/beta power decreases specific to word processing, no concurrent increases in gamma power were evident, neither in MEG nor in the more spatially resolved iEEG data. This absence of a significant effect does not exclude the possibility of a colocalized gamma power increase beneath the statistical threshold. However, the spectral tilt hypothesis does predict a strong co-occurrence of low-frequency decreases and high-frequency increase; thus, this single dissociation (effect in alpha/beta but no effect in gamma) is violating a key prediction of the spectral tilt hypothesis.

### Subsequent memory effects

Subsequent memory effects (SMEs, i.e., effects of memory encoding) were investigated in two time-frequency windows in which material-specific effects were evident: alpha/beta (8–20 Hz, 0.3–1.5 s) and gamma power (50–90 Hz, 0.3–1.0 s). Note that this selection of frequency-time windows exhibiting significant material-dependent power changes does not bias the finding-memory effects or interaction, as these are statistically independent contrasts. Additionally, to alpha/beta and gamma power changes, theta power changes were investigated in a third time-frequency window from 2 to 5 Hz and from 1.0 to 1.5 s based on prior studies [33,46]. We were specifically interested in interactions of memory and material, i.e., whether SMEs in specific frequency bands vary with material.

**Fig 4:**
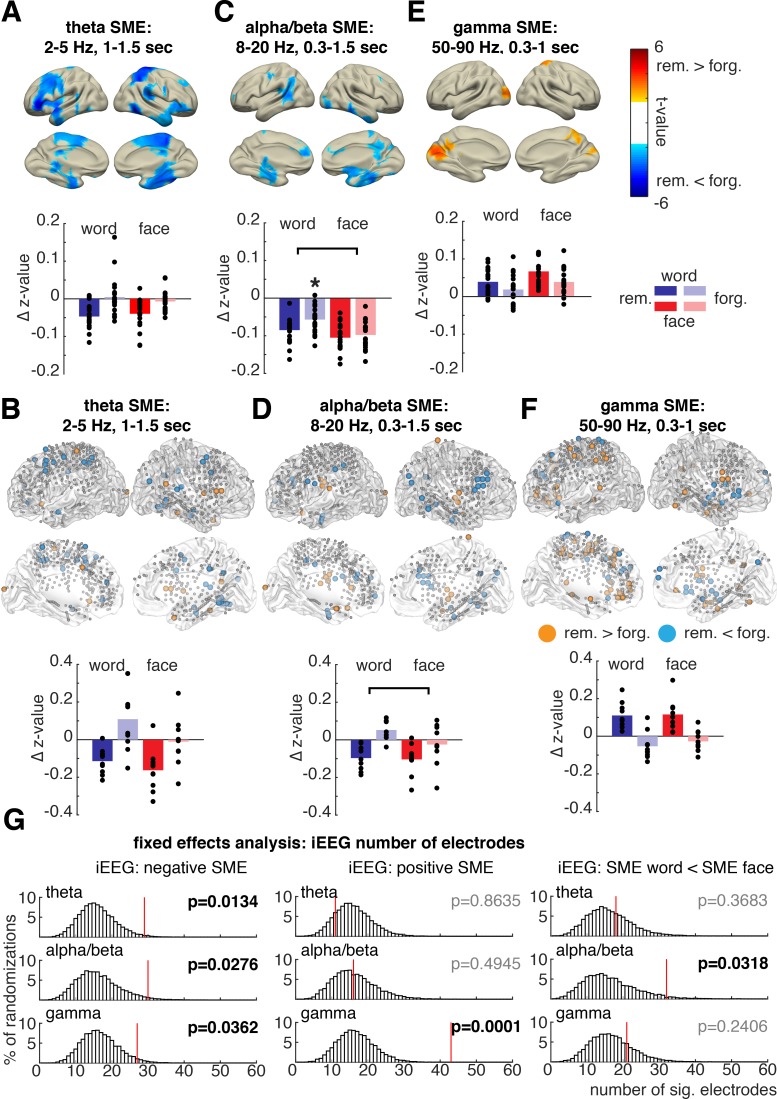
Material-independent and material-dependent SMEs. SMEs (remembered [“rem.”]-forgotten [“forg.”]) were analyzed in three time-frequency windows of interest. (A, C, E) Significant source clusters (*p*_corr_ < 0.05) for main effects of memory (SMEs irrespective of material). (B,D,F) iEEG electrodes exhibiting significant (*p* < 0.05, uncorrected) positive or negative main effects of memory are highlighted in orange or blue, respectively. Bar plots on the right show average normalized power for all condition averages for the respective MEG source cluster or iEEG electrodes plotted to the left, respectively. Black dots show the respective single-subject averages. Asterisks mark significant interaction effects (*p* < 0.05). (G) Results of a fixed-effects permutation analysis including all iEEG electrodes across all patients. The number of significant (“sig.”) electrodes in the data exhibiting a significant effect is highlighted in red relative to the distribution of significant electrodes in randomly shuffled data. Data and scripts underlying this figure are deposited here: https://osf.io/3csku/. iEEG, intracranial electroencephalography; MEG, magnetoencephalography; SME, subsequent memory effect.

To parallelize analysis of iEEG data to the analysis of MEG data, a two-stage procedure was used. In a first stage, ROIs (in MEG) or electrodes of interest (iEEG), which showed a main effect of memory (i.e., SMEs independent of material), were identified. In MEG, these ROIs were source clusters exhibiting significant main effects of memory; in iEEG, we selected electrodes with uncorrected *p* < 0.05 main effects of memory (similar to prior analysis of material-dependent changes). In the second stage, the data within these SME ROIs were tested for material × memory interactions. Notably, this procedure does not artificially inflate statistical power for finding interactions—it slightly biases the results against finding interactions, since only ROIs with a tendency for showing SMEs in the same direction across materials are considered. This procedure can readily be applied to MEG as well as to iEEG data, which is not trivial, since locations of electrodes vary from patient to patient, whereas MEG data provide whole-brain coverage in every subject. This analysis allows for random-effects analysis of memory × material interactions in iEEG data at the expense of limiting analysis to electrodes and patients exhibiting significant main effects of memory. Since this selection may theoretically distort the findings, an additional fixed-effects analysis (FEA) was run including all electrodes combined across all patients (657 electrodes, Fig 4G). The FEA tested whether the number of “significant” (i.e., uncorrected *p* < 0.05) electrodes across all patients exceeded the number of “significant” electrodes in a randomly permuted sample. This fixed-effect permutation analysis omits spatial clustering. iEEG data, in contrast to MEG data, only allow spatial clustering with strong limitations: spatially neighboring electrodes are more likely belong to one patient, magnifying the problem of outliers (i.e., single subject–driven effects). To validate the findings reported here, however, the results of an additional spatially cluster permutation statistic are reported in the S1 Text, which broadly replicate the results reported below (S3 Fig).

### Theta

In MEG, data decreases in theta power (2–5 Hz, 1–1.5 s) were observed for later-remembered compared to later-forgotten items (source *p* < 0.001, t-sum = −3,446.8; Fig 4A). Theta power decreases spanned a temporo-cortical network including inferior frontal, parietal, and temporal regions with a peak in the left inferior frontal gyrus (t_19_ = −5.82, MNI −56, 40, −10). This negative theta SME did not differ between words and faces (interaction memory × material: t_19_ = −1.02, *p* = 0.32).

These effects were paralleled in the iEEG data: significant decreases in theta power were observed for later-remembered compared to later-forgotten items (i.e., negative SME) in the same time-frequency window (2–5 Hz, 1–1.5 s, FEA: 29 electrodes, *p*_corr_ = 0.0134, Fig 4G). No significant positive SME—i.e., increase in power for remembered versus forgotten—was found (FEA: 11 electrodes, *p*_corr_ = 0.66). In electrodes selected for exhibiting negative theta SMEs, no difference in SMEs depending on material across patients was evident (Fig 3B; analysis including 10 patients, 1–6 electrodes per patient, *p* = 0.13, t_9_ = −1.66). In the FEA across all electrodes, this result was replicated; no significant material-dependent difference of SMEs was found (FEA: SME words < SME faces: *p* = 0.37, 18 electrodes Fig 4G, SME words > SME faces: *p*_corr_ = 0.75, 12 electrodes). Together, we observed the same pattern of results in MEG and iEEG. The data indicate that decreases in theta power predict memory formation independent of material.

### Alpha/Beta

In the MEG data, decreases in alpha/beta power (8–20 Hz, 0.3–1.5 s) predicted later memory (source-level *p* = 0.005, t-sum = −2,092.4). This negative SME was localized in areas encompassing typical encoding relevant regions: left superior frontal, inferior-medial temporal areas including the left hippocampus (peak right inferior temporal, t_19_ = −4.75, MNI: 74, −40, −20; Fig 4C). Importantly, the alpha/beta power SMEs significantly differed between faces and words, as indicated by a significant material × memory interaction (*p* = 0.020, t_19_ = −4.38). Alpha/beta SMEs were significantly stronger for words in contrast to faces.

The same pattern of effects was evident in the iEEG data. Significant decreases in alpha/beta power were related to successful encoding across electrodes (FEA: alpha/beta: *p*_corr_ = 0.028, 30 electrodes, Fig 4G); no significant increase in alpha/beta power during memory formation was found (FEA: *p*_corr_ = 0.49, 16 electrodes, Fig 4G). Across patients with electrodes showing negative alpha/beta SME, a similar significant difference in SMEs was evident as in MEG: here, again, a significant material × memory interaction was observed (*p* = 0.0027, t_8_ = −4.28, 9 patients, 1–7 electrodes per patient), which was driven by stronger power decreases for later-remembered words compared to later-forgotten words (Fig 4D). The same significant difference in SMEs between words and faces was obtained for alpha/beta power decreases across all electrodes (FEA: words < faces: *p*_corr_ = 0.032, 32 electrodes, SME words > SME faces: *p*_corr_ = 0.66, 13 electrodes, Fig 4G).

These stronger negative alpha/beta SMEs for words than for faces in MEG and iEEG data suggest a specific role of alpha/beta power decreases during memory formation for verbal material. This result further demonstrates that decreases in low-frequency power do not uniformly contribute to later memory, since this interaction effect was specific to the alpha/beta band but not evident in the theta range.

### Gamma

In the gamma time-frequency window (50–90 Hz, 0.3–1 s) a significant positive SME—i.e., increases for later-remembered items—emerged in the MEG data localized to occipital and parietal regions (Fig 4E, *p* = 0.048, t-sum = 479.06). The peak of the source was located in left superior occipital gyrus (max t_19_ = 4.34, MNI −14, −90, 10), spanning typical regions involved in visual processing: left calcarine gyrus, cuneus, lingual gyrus, and occipital superior and middle gyrus. These gamma power changes in visual areas did not vary with material (interaction *p* = 0.42, t_19_ = −0.81).

In accordance with MEG results, a significant number of electrodes showed significant gamma power increases related to memory formation in iEEG data (FEA: *p*_corr_ = 0.0001, 43 electrodes, Fig 4G). Contrary to the MEG results, a small but significant number of electrodes showed a decrease in gamma power for later-remembered items (FEA: *p*_corr_ = 0.036, 27 electrodes, Fig 4G). This negative gamma SME could have been missed in MEG because of the limited spatial resolution and overall slightly worse signal-to-noise ratio in high frequencies or could be a false positive in the iEEG data because of the inflated statistical power of the FEA. Concerning the material specificity of memory effects, however, similar results as in MEG data were again observed: gamma power SMEs did not vary with material (interaction material × memory *p* = 0.39, t_9_ = 0.91, 10 patients with 2–13 electrodes, Fig 4F). Similarly, no significant influence of material on SMEs was found in the FEA for the gamma frequency range (FEA: SME words < SME faces: *p*_corr_ = 0.24, 21 electrodes, SME words > SME faces: *p*_corr_ = 0.75, 13 electrodes, Fig 4G). Results from both iEEG and MEG showed that increases in gamma power index successful memory formation independently of material, thus mirroring the results of theta power.

To conclude, fixed-effects and random-effects analysis of iEEG data fully replicates the findings obtained in MEG: negative alpha/beta SMEs vary with material; positive gamma SMEs and negative theta SMEs are found irrespective of material. To further show the frequency specificity of reported SMEs, S5 Fig shows the average power spectra in each selected ROI in iEEG and MEG data. This frequency-specific pattern of SME material dependency demonstrates that changes in specific frequency bands index specific processing demands during encoding.

### Latency differences between gamma and theta SMEs

**Fig 5:**
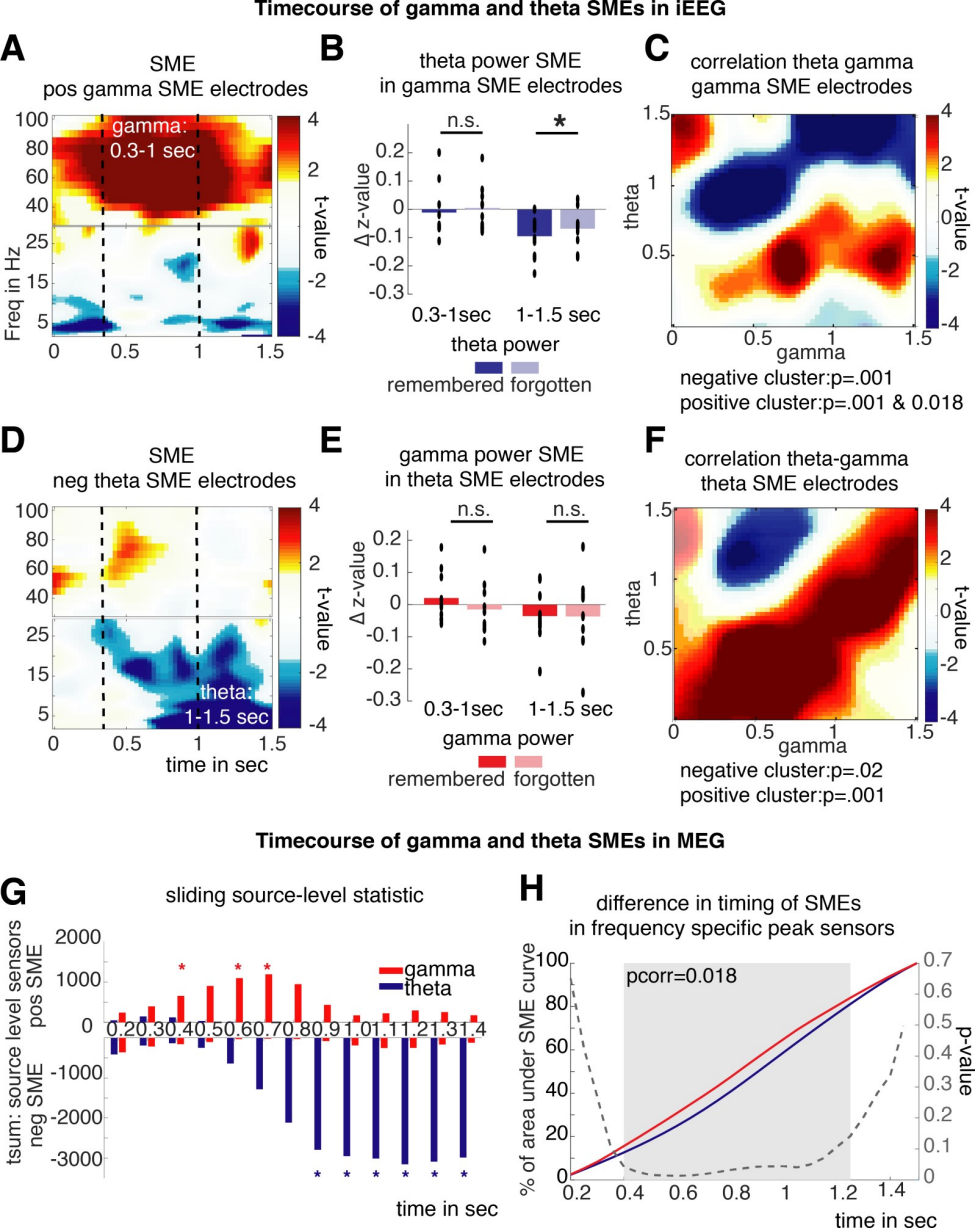
Timing differences of theta and gamma SMEs. (A–D) Differences in timing in iEEG data. Time-frequency (“Freq”) plots depict t-values of SMEs in iEEG electrodes exhibiting a significant positive (“pos”) SME in the gamma frequency range or a significant negative (“neg”) SME in the theta frequency range, respectively. Dashed lines highlight the time windows of gamma SMEs (0.3–1 sec) and theta SMEs (1.0–1.5 sec). (B) Bar plots show average theta SMEs in electrodes defined by gamma SMEs and gamma SMEs in electrodes showing theta SMEs for the earlier gamma and later theta time window. Black dots show the respective single-subject averages; black asterisks mark significant differences (*p* < 0.05). (E, F) Differences in timing of theta and gamma SMEs in MEG. (C and F) Plots show clusters of significant correlations between gamma and theta power time courses for every latency combination; significant clusters are highlighted by transparency. (G) Bars show the t-sum across significant negative and positive source-level sensors of gamma and theta SMEs calculated on power averages in a 300-ms windows with 100-ms increments. Red and blue asterisks mark significant positive gamma SMEs of negative theta SMEs at the given time point, respectively. (H) For each MEG dataset, the 5% source-level sensors were identified that exhibited the highest frequency-specific SMEs across 0.2–1.5 s. The average area under the SME curve reached at each time point in these frequency- and subject-specific ROIs is plotted for gamma and theta SMEs; the gray area highlights the significant cluster (random cluster permutation) in which gamma SMEs significantly precede theta SMEs. Data and scripts underlying this figure are deposited here: https://osf.io/3csku/. iEEG, intracranial electroencephalography; MEG, magnetoencephalography; n.s., no significant difference; ROI, region of interest; SME, subsequent memory effect.

The analyses described above demonstrate a functional dissociation of decreases in low-frequency and increases in higher-frequency power, suggesting that these changes do not reflect a single process. Specifically, alpha/beta power decreases varied with material during memory formation, whereas theta power decreases and gamma power increases did not. However, power decreases in theta and power increases in gamma did show a similar pattern of SMEs—i.e., both accompany memory formation independent of material. Therefore, in a further analysis, we focused on the relationship between these two frequency bands. More specifically, we asked whether theta power decreases and gamma power increases occur in the same regions and at the same time. In iEEG data, we calculated gamma SMEs in electrodes exhibiting significant negative theta SMEs and vice versa. Power spectra from all selected electrodes were averaged in each subject and entered into a dependent *t* test (with *N* subjects as random variable). In electrodes exhibiting a significant positive gamma SME, no significant negative theta SME was evident in the early time window, in which gamma power changes were observed (Fig 5A and 5B, 2–5 Hz, 0.3–1.0 s, t_9_ = −1.162, *p* = 0.275). However, in a later time window, theta power significantly decreased for remembered versus forgotten items (2–5 Hz, 1.0–1.5 s, t_9_ = −3.137, *p* = 0.0120, Fig 5B). This result indicates that theta and gamma SMEs can be located at the same regions but seem shifted in time (i.e., gamma SME precedes theta SME).

This same analysis was repeated, now investigating whether gamma SMEs can be found in electrodes exhibiting significant theta SMEs (Fig 5C). Again, no significant gamma SMEs were evident at the same time window when theta SMEs were predominant (50–90 Hz, 1.0–1.5 s: t_9_ = 0.058, *p* = 0.958, Fig 5D). However, in the early time window, gamma SMEs were observed but did not reach significance (50–90 Hz, 0.3–1.0 s, t_9_ = 1.785, *p* = 0.11, Fig 5D). This result agrees with the preceding analysis in suggesting that theta and gamma SMEs may overlap spatially but can be temporally dissociated.

To formally assess whether negative theta SMEs and positive gamma SMEs are indeed shifted in time, theta (2–5 Hz) and gamma power (50–90 Hz) time courses were correlated across trials for every combination of time points from 0 to 1,500 ms post stimulus in electrodes selected based on negative theta SMEs or positive gamma SMEs, respectively. As theta and gamma SMEs exhibit a negative relationship, the analysis regarding timing differences is focused on the theta latency and gamma latency combination at which a significant negative correlation is evident. A cluster permutation test was used to investigate whether correlations are significantly positive or negative across electrodes at different theta–gamma time course combinations in theta SME electrodes as well as gamma SME electrodes. This analysis revealed two significant clusters in theta SME electrodes (Fig 5F negative cluster: *p*_corr_ = 0.02, positive cluster: *p*_corr_ = 0.001), as well as in gamma SME electrodes (Fig 5C negative cluster: *p*_corr_ = 0.001, two-positives cluster: *p*_corr_ = 0.001 and 0.018). Importantly, the negative clusters were shifted in time: theta power around 1,000–1,500 ms correlated consistently negative with earlier gamma power (approximately 300–900 ms) in theta electrodes, and a similar time lag was evident in gamma electrodes. We also observed positive correlations along the diagonal of the time-by-time matrix, i.e., for non-time-lagged correlations. There are several possible causes for this positive correlation, such as event-related potentials (ERPs), a coupling of theta and gamma activity, baseline shifts, etc. Although the present analysis cannot disentangle the cause of these positive time-locked correlations, they are further contradicting a spectral tilt explanation of SMEs, as a tilt explanation of SMEs would predict a negative correlation along the diagonal. Together, this analysis shows that positive gamma SMEs and negative theta SMEs may be related but are shifted in time, which does not fit with a tilt-based explanation of memory-related power changes.

Intracranial EEG data are inherently restricted to the clinically defined implantation sites, and therefore, one cannot exclude that theta and gamma effects overlap in areas where no electrodes were implanted. This limitation does not exist for whole-brain recording methods like MEG. Therefore, we investigated whether the general timing of theta and gamma SMEs on a whole-brain level followed our previous findings. To provide a more time-resolved depiction of SMEs, a cluster permutation statistic on the source data was calculated in sliding time windows of 300 ms (with increments of 100 ms) in the gamma frequency band (50–90 Hz) and the theta band (2–5 Hz). In Fig 5E, the respective positive and negative t-sums across all significant source sensors are plotted. From this plot, it is evident that positive gamma SMEs appear earlier than negative theta SMEs.

To formally quantify these latency differences between gamma SMEs and theta SMEs, we calculated the percentage of the area under the curve at each time point by cumulating the SME curve across time for each subject and frequency-specific peak source sensors (5% absolute highest gamma or theta SME summed across 0.2–1.5 s). This analysis allows for an unbiased contrast of latency between SMEs of the two frequencies. Each frequency starts at the same point (0%) and finishes at the same point (100%). Analogously, this analysis is like pitching two horses against each other in a race and measuring the distance covered at each time point to see which one is faster. This area-under-the-curve method is more sensitive to detect differences in timing than classical peak latency comparisons [47] and is not affected by differences in time resolution (e.g., smoothness between gamma and theta SMEs; see S8 Fig). In Fig 5F, the area under the curve for negative theta SMEs and positive gamma SMEs is shown for each time bin from 0.2 to 1.5 s. The latency curve for positive gamma SMEs is leading in respect to the theta SME curve, indicating gamma SMEs earlier in time compared to theta SMEs. A cluster permutation statistic permuting the individual latency curve across subjects showed a significant difference in the timing of gamma and theta SMEs (cluster permutation *p*_corr_ = 0.018). Note that this result remains stable also when employing different selection criteria (i.e., no thresholding of SME curves and no preselection of peak effect sensors, see S9 Fig).

Together, these results illustrate that low-frequency negative SMEs can be colocated to similar regions where positive SMEs in higher frequencies are observed. However, the SMEs in the gamma and theta frequency bands are temporally dissociated, with increased gamma power preceding decreases in theta power during the encoding of subsequently remembered items. This latter result indicates that the two frequency bands index different cognitive processes that occur at different time points in the service of memory.

### Fitting spectral tilts: SMEs in spectral slopes and residual power

To further rule out that the reported memory effects can be explained by a simple shift in the tilt of the frequency spectra, we used a robust regression to fit slopes to the average power spectrum across trial in each condition of every MEG and iEEG dataset. Since prior literature used varying frequency spans to fit the tilt (i.e., slope) of the spectrum, slopes were calculated based on three different frequency ranges (2–30 Hz, 30–90 Hz, 2–90 Hz). Based on the fitted slope and offset, the residual power spectrum was calculated by subtracting the fitted regression line. Interestingly in both datasets, MEG and iEEG, there were task-related differences between estimated slopes. These differences, however, highly depended on which frequency bands the fit was based on (see S2 Text and S6 and S7 Figs). Investigating SMEs in residual frequencies showed stable SMEs in the respective frequency bands, even after subtraction of the condition-specific frequency tilt (S6 and S7 Figs), except gamma SMEs in MEG data. This pattern of results demonstrates that a simple shift in the power spectrum might contribute to reported SMEs but cannot completely explain the full pattern of results.

## Discussion

The present results support the hypothesis that power changes in different frequency bands during memory encoding represent distinct fingerprints of differentiable encoding processes. In line with prior studies, decreases in theta and alpha/beta power and increases in gamma power predicted subsequent memory. Unlike the reported uniform decreases in low-frequency power and increases in high-frequency power predicting memory formation [8,20], the present results demonstrate that theta, alpha/beta, and gamma power changes during encoding exhibit dissociable characteristics inconsistent with the frequency tilt hypothesis: (1) areas involved in word and face processing exhibit material-dependent decreases in alpha/beta power, whereas concurrent increases in gamma power were evident only in posterior sensory-processing regions but not in left lateralized areas responsive to word processing; (2) negative alpha/beta SMEs depend on material and are stronger for words than for faces, whereas negative theta SMEs and positive gamma SMEs do not depend on material; and (3) negative theta SMEs and positive gamma SMEs differ in timing, with gamma SMEs preceding theta SMEs. A particular strength of this study is that we show the same pattern of results in two independent datasets recorded in MEG and in iEEG. The reported dissociations are therefore present at different spatial scales and independent of how the signal is being measured.

Our results replicate the commonly found pattern of low-frequency power decreases and high-frequency power increases during memory encoding. However, in-depth analysis of this pattern revealed functionally, spatially, and temporally distinct signatures exhibited by different frequency bands. A simple analysis of time-averaged power spectral density [11,17], as proposed for analyzing spectral tilts in the data, would have concealed the diverse frequency- and time-specific patterns underlying memory formation. That said, summarizing complex changes in the power spectrum in simpler metrics like tilt or broadband shifts clearly offers useful tools for characterizing overall brain states [48,49], especially regarding pathological states or age-related changes [11]. However, these metrics have a risk of concealing more fine-grained temporal and spectral dynamics underlying complex cognitive processing, like memory formation. To understand the neural dynamics involved in cognition, the explanatory value of spectral tilts or broadband shifts remains therefore limited. The specific relation of alpha/beta decreases to the encoding of words and the differences in timing of theta and gamma SMEs demonstrate that decreases in lower frequencies and increases in higher frequencies are not a uniform, ubiquitous marker of memory encoding. Instead, they index separable processes, which likely have explanatory value to understand transformation of experiences in durable memory traces and are key for developing frequency-specific and oscillation-informed stimulation approaches [50].

Alpha/beta power in both iEEG and MEG specifically varied depending on the type of material to be encoded. Alpha/beta SMEs were stronger for the encoding of words in contrast to faces, i.e., material differing with respect to semantic-processing properties. This interaction is specific to the alpha/beta band and thus cannot be explained from a spectral tilt perspective. If successful encoding of words is simply related to a “flatter” tilt of the power spectrum, we should also see similar SMEs specific for words in the theta band and mirroring SMEs in higher frequencies. However, in both datasets, MEG and iEEG, theta SMEs did not show the same interaction pattern as alpha/beta SMEs. An additional analysis fitting spectral tilt and analyzing SMEs in the tilt-corrected power spectra revealed that across different tilt fits, SMEs largely remained stable in the tilt-corrected power spectra (see S6 and S7 Figs). These findings together show that a simple tilt model cannot explain the reported memory-related power changes.

The present findings are consistent with frameworks that propose specific roles of different frequency bands, i.e., spectral fingerprints of cognitive processing [26,27] of different frequency bands to multiplex content-specific memory processes [51]. The dissociable roles of gamma and alpha/beta band oscillations have been studied in attention tasks [12,52]. Gamma oscillations in general have been hypothesized to play an important role in local bottom-up sensory processing, whereas changes in alpha/beta oscillations have been related to long-range cortical communication and top-down processing [12,53,54]. This view of bottom-up and top-down processes is also in line with the current findings: gamma power increases were confined to occipital sensory areas and evident early after the stimulus, whereas decreases in alpha/beta and theta power were evident in widespread distributed cortical and medio-temporal regions in a later time window, reflecting possibly higher-level processes involving top-down control. The pattern of distinct spectral fingerprints in the present data concurs with cognitive memory models like Tulving’s SPI model [29] or PIMMS [30], which hypothesize memory encoding not as one monolithic process but highlight the role of different subprocesses. Our results show dissociable spectral fingerprints, which resemble commonly assumed stages in these models: perceptual, semantic, and episodic processes.

Previous behavioral research has shown that words are especially well remembered if semantically processed [39], whereas memory for unfamiliar faces does not benefit from semantic encoding strategies [40]. We demonstrate that decreases in alpha/beta power specifically index encoding of verbalizable material. This finding agrees with previous studies showing that decreases in alpha/beta power are specifically related to semantic encoding [36,37,55]. Beta oscillations in particular have been connected to language and semantic processes [56,57]. On a more general level, alpha/beta decreases have been linked to cortical information processing [58], or to distributed top-down networks [59,60]. This view is also in line with prior studies that reported complex item-specific representations being coded in the alpha/beta frequency range [61–63]. Because of these hypothesized features, alpha/beta oscillations might be a specific marker of the neural mechanisms behind the processing of distributed semantic features [64]. In light of the present results and prior studies, alpha/beta decreases during memory formation seem to be specific spectral fingerprints of semantic processing. The higher memory performance for words compared to faces on a cognitive level also highlights the important role of the semantic system as the main route to episodic memory [65]. The specific relationship of alpha/beta power decreases to the encoding of verbalizable material is a promising first step to a deeper understanding of the interaction of semantic and episodic memory.

Decreases in theta power and increases in gamma power related to subsequent memory are unspecific for the material that is to be encoded. Theta power decreases were spanning widespread cortical areas including MTL, frontal lobe, and temporal and parietal areas, resembling the core memory network [66] and matching prior results [8,34]. Changes in the gamma band during encoding were specifically located in cortical areas involved in sensory-visual processing, in line with previously reported SMEs in visual cortex [67], and in ventral occipitotemporal regions [8,23]. The insensitivity of the theta and gamma power changes to material and the general prominence of theta power changes in memory-encoding studies suggest a role of theta decreases as a marker of MTL-related memory-encoding mechanisms independent of how stimuli are processed.

A careful analysis of the temporal dynamics of the gamma and theta SMEs revealed dissociable time courses of these effects. SMEs in gamma power preceded SMEs in theta (Fig 5). Such a latency difference is in line with the view of gamma power indexing early perceptual processing stages, whereas theta power changes reflect later MTL-related memory processes. Theoretical models of memory put these two computations usually at opposing ends of the processing cascade [29,30]. Methods that average power spectra across time are unable to detect these differences [17,20] and lose a major advantage of electrophysiological recordings, i.e., its high temporal resolution. Together, the timing difference between gamma and theta SMEs demonstrates that theta power decreases and gamma power increases reflect different neural processes, which occur in sequence during memory encoding.

Our findings show that low-frequency decreases and high-frequency increases do not necessarily overlap in time and space. In addition to the differences in timing between gamma and theta SMEs, gamma power increases did not consistently occur in the same brain regions as alpha/beta power decreases during processing of words. Whereas gamma power increases co-occurred with alpha/beta power decreases in occipital/posterior regions [68], no such colocalization was evident in left lateralized areas involved in semantic processing. Consequently, if the analysis in our study was limited to increases in broadband gamma power changes, a common practice in iEEG analysis [9, 21–25], the prominent changes in alpha/beta power related to word processing would have been missed. Our findings are exemplary in demonstrating that limiting analysis to very narrow parts of the time-frequency spectrum restricts the potential insights that can be drawn from the data.

Stimulus-related gamma power changes are a currently debated topic. There is evidence that stimulus-related gamma power changes can be caused by two different mechanisms: a broadband activity change potentially indicating increased multiunit activity [9,16,69,70] or narrow band power changes indicating a true oscillatory response [3,71]. In the present analysis, we did not tackle this problem. All reported analysis is focused on relative broadband activity averaged from 50 to 90 Hz. The focus of the presented results is on whether low- and high-frequency changes during memory formation are indicating different processes or a uniform frequency tilt. However, an in-depth analysis of gamma changes during memory formation could reveal insights in whether gamma changes can also be broken down into different processes.

A unique aspect of the present study is that oscillatory brain activity was measured with two modalities and in two separate subject groups. Intracranial recordings in patients with epilepsy and MEG recordings in a healthy student population exhibited concordant pattern of results. Importantly, both MEG and iEEG come with their own specific strengths and limitations [42,43]. MEG sensors, for instance, have different noise levels as a consequence of the sensors being mounted in a dewer, which results in different distances of sensors from brain tissue and different susceptibility to movement artifacts. These problems could affect signal-to-noise ratio at certain brain areas and frequency ranges, making it, for example, difficult to detect gamma effects in frontal regions. This problem does not exist with iEEG, in which electrodes are implanted directly in the brain tissue, thus allowing one to record electrophysiological activity in all frequency bands with high spatial resolution. MEG has two major advantages over iEEG: (1) whole-head coverage in all subjects and (2) activity recorded in healthy participants rather than in a patient population. Although often seen as the gold standard for electrophysiological studies in humans, iEEG data inherently suffer from the fact that the data are recorded in a nonhealthy brain, possibly confounded with changes in frequency spectra, epileptogenic changes, and artifacts [72,73]. Here, we sought to combine the two methods in a complementary manner in order to overcome their respective limitations. Indeed, the tight overlap between iEEG and MEG results described here lends confidence in both results in that the iEEG data verify the MEG source reconstruction, and the MEG data alleviate concerns about the generalizability of the iEEG findings. MEG and iEEG results are remarkably similar despite differences in the sample (homogenous student sample in MEG versus a more diverse patient sample in iEEG), differences in memory performance, and slight differences in paradigm timing (see Fig 1A). Prior studies have combined M/EEG and iEEG, albeit typically in very small samples [32,33]. The present study is, to the best of our knowledge, the first to combine MEG and iEEG data of comparable sample sizes to study oscillatory processes in memory encoding. The present results therefore offer a unique validation of the commonly implied interchangeability of MEG and iEEG results.

An open question that remains is why decreases in theta power are involved in memory encoding in general, whereas alpha/beta power decreases are specifically involved in the encoding of semantically meaningful material. Arguably, both processes involve the integration of representations coded in widespread neural networks. Interestingly, power decreases, which indicate a decrease in local synchronization, are often found to co-occur with increases in long-range phase synchronization [8,74–76]. Therefore, decreases in power (i.e., local connectivity) might be a prerequisite for the formation of large-scale, fine-grained connectivity vital for distributed neural representations. The present findings thereby open up important follow-up questions concerning the relationship of local power decreases and network connectivity.

To conclude, we recorded electrophysiological activity during memory encoding in two complementary modalities, MEG and intracranial EEG. Our data provide evidence that the reported decreases in low-frequency power and concurrent increases in high-frequency power during memory encoding are not general markers of neural activity. Instead, the different responses in low- and high-frequency bands reflect spectral fingerprints of dissociable memory-encoding processes. Considering interactions of different memory system networks, the specific relationship of alpha/beta power changes to semantic processing opens a window to the relationship of semantic and episodic memory, which has, as yet, not been well studied. Speculatively, interactions of alpha/beta and theta networks might specifically mark the interplay between semantic and episodic memory, which crucially shapes human memories [29,30].

## Material and methods

### Ethics statement

The protocols adhered to the Declaration of Helsinki and were approved by the ethics committee of the University Konstanz, University of Birmingham, the Friedrich Alexander University of Erlangen, and National Hospital for Neurology and Neurosurgery (ERN_14–0651, 142_12, and 10/H0715/63, respectively). All participants gave their written, informed consent.

### Participants

Thirty-two volunteers participated in the MEG experiment (compensated with course credit or €10/hour). Data from 11 participants were excluded because of low trial numbers in one of the conditions (minimum 30 trials) after rejecting MEG artifacts and trials with early-response button presses. One additional dataset was excluded because of an erroneous head-shape digitization, resulting in a sample of 20 subjects (mean age = 23.5 y, range 18–33 y, 6 male). All subjects were right-handed, spoke German as their native language, reported no history of neurologic or psychiatric disease, and had normal or corrected-to-normal vision.

An additional 22 patients with pharmaco-resistant epilepsy who were implanted with intracranial electrodes for diagnostic purposes volunteered to participate in a matching memory-encoding study. Data of 17 patients were recorded at the University Hospital Erlangen, and data of five patients were recorded in cooperation with University College London at the National Hospital for Neurology and Neurosurgery. Data of eight patients were excluded from later analyses because of either technical problems during recordings, left-handedness, or insufficient memory performance. In the remaining dataset of 13 patients (mean age = 35.54 y, range: 20–60 y, 3 male), eight patients were native German speakers, four were native English speakers, and one spoke Slovenian. Word material and instructions were translated accordingly.

### Material

Word material was drawn from the MRC Psycholinguistic Database [77], translated into German/English/Slovenian depending on the respective participant (264 words during experiment, additional 12 words for practice trials). Neutral unfamiliar faces (264 faces during experiment, additional 12 faces for practice trials) were drawn from several face material databases ([78] and pics.stir.ac.uk). All face stimuli had an emotionally neutral expression and were presented in grayscale on a black background. Use of word and face material during encoding and as new material during recognition was counterbalanced across participants.

### Procedure

Every participant completed two task blocks: one block of word encoding and recognition and one block of encoding and recognizing unfamiliar faces (order counterbalanced across participants). During the whole experiment, MEG or iEEG was recorded.

During the encoding phase, participants were instructed to judge each item presented for pleasantness on a 1–6 scale. The encoding phase was followed by a distractor task to prevent working-memory contributions to the recognition test. The distractor task was a variation of the inattentional blindness task as used in [79]. During the recognition phase, all previously shown items were presented randomly intermixed with new items, i.e., lures. Participants were instructed to provide confidence ratings ranging from 1, very sure old, to 6, very sure new. Prior to each phase, participants completed a short practice phase to familiarize them with the paradigm. In the MEG sample, responses were given using two response boxes with three buttons placed on the right and left side of the body; response-hand use was counterbalanced across participants. Because of the test setting in the hospital bed in the iEEG sample, response-hand use was not controlled, as flexible response-hand use was not always possible. Patients completed the same paradigm as healthy controls with small adaptions (a reduced number of trials, self-paced responses and additional breaks, see Fig 1A).

### Behavioral analysis

An ROC approach was used to analyze memory performance. A single-process unequal-variance model was fitted to the data to obtain bias-free measures of memory strength [80,81] and classify hits and misses relative to individually defined neutral response criteria for MEG/iEEG analysis (for details of fitting procedures, see [35,37]). In short, this approach assumes that memory strength can be modeled by separate normal distributions for new and old items. The distance d′ of the mean of these distributions yields a bias-free measure of memory strength. The model assumes that subjects respond with a certain confidence rating i whenever their subjective memory strength exceeds a certain criterion c_i_. The crossover of the distributions of new and old items represents the point of the neutral response criterion, as this point represents the memory strength that has an equal probability to be elicited by new and old items. An item that during recognition received a confidence rating i was classified as a hit if the corresponding estimated criterion c_i_ was higher than the individually estimated neutral criterion in the recognition block; otherwise, the trial was classified as a miss. As demonstrated previously, this procedure enhances signal-to-noise ratio by considering individual differences in the use of confidence ratings for hit-and-miss trial definition [35].

### MEG recording and processing

MEG was recorded with a 148-channel whole-cortex magnetometer (MAGNES 2500 WH, 4D Neuroimaging, San Diego, CA, United States) in a magnetically shielded room while participants were in a supine position. Data were continuously recorded at a sampling rate of 678.17 Hz and bandwidth of 0.1–200 Hz. The participants’ nasion, left and right ear canal, and head shape were digitized prior to each session with a Polhemus 3Space Fasttrack.

All analyses were carried out in MATLAB (The MathWorks, Natick, MA, USA) using the fieldtrip toolbox (www.ru.nl/fcdonders/fieldtrip, [82]). Data from encoding phases were epoched in trials −1.5 to 3 s around each item onset during encoding. Line noise was removed by a discrete Fourier transform filter. Idiographic artifacts (channel jumps, muscle artifacts, noisy channels) were excluded from further analysis by visual inspection. Infomax independent component analysis was applied to correct for residual artifacts (e.g., eyeblinks, eye movements, heartbeat-related activity, or tonic muscle activity). On average, 105.8 word-hit trials (SD = 18.7, range: 75–129), 51.6 word-miss trials (SD = 18.4, range 31–88), 85.8 face-hit trials (SD = 17.3, range: 39–111), and 71.8 faces-miss trials (mean = 71.8, SD = 18.0, range: 49–111) passed artifact correction. MEG sensor-level data were transformed into planar gradients for sensor-level analysis. This procedure emphasizes activity directly above a source, simplifying interpretation of MEG topographies [83]. Source analysis was carried out using a linearly constrained minimal variance (LCMV) beamformer [84], calculating a spatial filter based on the whole length of all trials. Individual structural MR images were aligned with the MEG sensor coordinates using NUT-MEG [85]. Individual single-shell head models [86] were constructed using structural MRIs of each participant. The brain space was divided in 10-mm grid voxels and normalized to the MNI brain using a warping procedure. Source time courses for each grid point were calculated and subjected to a wavelet analysis described below.

Data were filtered to obtain lower-frequency oscillatory power between 2 and 30 Hz using wavelets with a 5-cycle length; resulting time-frequency data were smoothed with a Gaussian kernel (FWHM 200 ms and 2 Hz) to account for interindividual differences and changes in time-frequency resolution across frequencies. To obtain higher-frequency oscillatory power in the gamma range (30–100 Hz), a multitaper approach was used with a 300-ms window and a spectral smoothing of ±10 Hz, resulting in the use of five tapers. Resulting data were z-transformed to the respective mean and standard deviation across time for every time-frequency bin separately for two different recording blocks of words and faces but not separately for hits and misses (e.g., [8]). Average mean and SD used for z-transformation are plotted in S4 Fig, showing that mean and SD across trials subtracts also the average 1/f characteristics of the mean signal.

### iEEG recording and processing

Intracranial data were recorded from subdural grid, strip, and depth electrodes (AdTech, recording system Deltamed, Natus or Nicolet, NicVue) referenced to a scalp electrode. The implantation scheme depended on the suspected epileptic foci and was therefore highly variable across patients (see Fig 1C). Locations of electrodes were determined using coregistered postimplantation MRIs and postimplantation CTs. Locations were then transformed to MNI coordinates by normalizing the postimplantation MRIs to standard MNI space using SPM8. Data were continuously recorded at different sampling rates (4 datasets: 512 Hz, 8 datasets: 1,024 Hz, 1 dataset: 4,096 Hz).

Data from encoding phases were epoched in trials from −1.5 to 3 secs around each item onset during encoding and downsampled to a sampling rate of 500 Hz to match sampling rate across datasets. Data were referenced to bipolar montages to obtain maximally focal spatial resolution [87]. To this end, each electrode was re-referenced to its neighboring electrode (for grid electrodes across the horizontal and vertical dimension). Coordinates of these bipolar “virtual” electrodes were calculated as located between the respective physical electrodes. Electrodes within or bordering areas later resected or identified as the epileptic foci were excluded from analysis. Data were carefully visually inspected by a trained neurologist and a second individual; electrodes with epileptogenic activity were excluded from analysis. Individual trials that exceed the mean range, variance, or kurtosis by more than 5 SDs were automatically rejected. Channels yielding fewer than 10 trials after artifact correction in any condition were rejected. This resulted in a dataset of 657 bipolar channels (of 926 recorded channels, mean = 51.85 per patient, SD = 24.37, range 9–88). The mean number of trials across channels and patients for word hits was 63.61 (SD = 10.44, range: 47.16–83.72), for face hits 47.84 (SD = 13.45, range: 19.15–68.70), for word misses 27.39 (SD = 10.40, range: 12.61–43.45), and for face misses 41.63 (SD = 15.56, range: 24.47–72). iEEG data were filtered to obtain oscillatory power, z-transformed, and smoothed using the same settings as MEG data.

### Statistical analysis: Memory and material effects

The study follows a 2 × 2 design with the factors memory (remembered versus forgotten) and material (face versus word). The analysis scheme of iEEG and MEG data differed as a result of the different nature of these two datasets.

MEG data were analyzed using a conventional repeated-measurements random-effects design. Task contrasts of interest were interaction effects and main effects of the 2 × 2 repeated-measurements design (i.e., power spectrum for material × memory). In order to stay within the fieldtrip cluster statistic framework, these contrasts were calculated using the cluster permutated t-contrasts. In each subject, power spectra at each sensor/source-level sensor were averaged across all trials for each cell of the 2 × 2 design matrix (word hits, word miss, face hit, face miss). Main effects were analyzed by contrasting the means across the respective cells using dependent t-contrasts (i.e., mean of word hits and word misses versus mean of face hits and face misses as main effect of material). Interaction effects were calculated by contrasting the material-specific SMEs (i.e., word hit minus word miss contrasted to face hit minus face miss). Averaging of cell-specific means prevents a potential biasing of main effects by trial-number differences across condition (i.e., more word-hit trials than face-hit trials). This analysis scheme of *t* tests for testing interaction and main effects is equivalent to a 2 × 2 repeated-measures ANOVA.

Statistical analysis of MEG data was carried out using the fieldtrip cluster permutation approach [88]. The cluster permutation test consists of two steps: First, clusters of coherent t-values exceeding a certain threshold along selected dimensions (time, frequency, electrodes/grid voxels) are detected in the data. Second, summed t-values of these clusters are compared to a null distribution of t-sums of random clusters obtained by permuting condition labels across subjects. This procedure effectively controls for type I errors due to multiple testing. For sensor analysis, 3D clusters (electrodes × time × frequency) were built by identifying neighboring time-frequency-channel bins involving at least two neighboring channels with a *p*-value below 0.01 (lower *p*-threshold to identify coherent clusters in higher-dimensional clustering). For source-space analysis, clusters were formed across the spatial dimension (*p*-level threshold 0.05).

iEEG electrodes across the whole patient sample covered widespread brain areas; however, the varying electrode implantation scheme in each patient impeded a similar random-effects analysis as in MEG. iEEG analysis was restricted to the two time-frequency windows identified in MEG analysis (alpha/beta 8–20 Hz, 0.3–1.5 s, gamma: 50–90 Hz, 0.3–1 sec) or a priori defined (theta 2–5 Hz, 1–1.5 s). A 2 × 2 ANOVA (memory × material) was calculated on the single trials in each single electrode. Concordance of iEEG and MEG results was tested using χ^2^ tests of independency. Calculating a χ^2^ test requires two different categorical variables in one single population (here: iEEG effect direction and location relative to MEG-source clusters). The overlap of iEEG and MEG effects was consequently estimated by counting the number of iEEG electrodes in each significant MEG-source cluster showing an uncorrected *p* < 0.05 negative, positive, or no difference. These absolute numbers of electrodes in each MEG-defined region was used to construct a contingency table, and a χ^2^ test of independence was employed to assess statistical significance of the dependency of MEG and iEEG effects. In a control analysis, χ^2^ test of independence was additionally calculated employing a more liberal categorization of iEEG electrodes based solely on direction of effects (positive or negative difference, see S2 Fig).

To elucidate differences in SMEs (i.e., interaction of material × memory) in iEEG data in a group random-effects manner, all electrodes showing a significant main effect of memory in a frequency band of interest were averaged in each subject and subjected to the same random-effects repeated-measurement ANOVA analysis schema as MEG data. This random-effects group analysis of an interaction was restricted to preselected electrodes exhibiting a main effect of memory. This preselection is no circular analysis (no “double dipping”), as interaction effects are independent of main effects.

To further ensure that the preselection of memory-effect electrodes is not missing effects, an additional FEA combining all electrodes across all patients was carried out. To this end, a random distribution of the number of electrodes showing a negative or positive main or interaction across the whole patient/electrode sample was estimated by randomly shuffling the trial labels (word–face, remembered–forgotten) in each subject 10,000 times and calculating the number of significant electrodes in each permutation. If the number of electrodes exhibiting a significant main or interaction effect in the data was observed in less than 5% of permutation, the pattern of results was regarded as significant.

### Analysis of latency differences

For analysis of timing differences of gamma and theta SMEs in iEEG, we employed a correlation analysis. For this analysis, theta (2–5 Hz) and gamma power (50–90 Hz) were correlated across trials in selected electrodes for every time point × time point combination by calculating Fisher z-transformed Pearson’s correlation coefficients. To assess whether and at which time point combinations are consistently positive or negative correlations evident across selected electrodes, a cluster permutation test was applied. As a first-level statistic, t-values were calculated testing correlations against zero. These t-values were then summed across coherent 2D cluster in time × time space. To assess the significance of these t-sum values, this procedure was repeated 1,000 times for randomly shuffled trials to generate a distribution of coherent 2D clusters under the null hypothesis. The summed t-values of coherent clusters from the real data were then compared against the summed t-values of coherent clusters from the randomized data.

For analysis of timing differences of gamma and theta SMEs in MEG, we employed an area-under-the-curve analysis, which has been shown to be more reliable in finding latency differences compared to peak latency analysis [89]. First, a t-contrast was calculated for theta and gamma power for all remembered versus forgotten trials in each subject for each source-level sensor at each time bin between 200 and 1,500 ms. Second, these t-value time courses were thresholded at zero; for gamma SMEs, all negative t-values were set to zero (as timing of positive SMEs was of interest), and vice versa for theta SMEs (all positive t-values were set to zero). Third, frequency-specific peaks were identified by summing these thresholded t-value time series across the whole time series and selecting the 5% of sensors with the highest absolute t-sum. Fourth, the t-value time series was averaged across the peak sensors in each subject, and the area under the SME curve was calculated by integrating across the time dimension (i.e., summing at each time point the t-values up to this time point relative to the t-sum across all time bins). These specific thresholding and sensor-selection criteria were employed to allow for a conservative estimation of latency differences. Running the timing analysis in the ROIs reported in Fig 4 biases the analysis to show earlier gamma and later theta effects. This necessitates an approach in which sensors are selected based on criteria that render any timing comparison between the two frequency bands fair (i.e., unbiased). Important points to be considered are that (1) the selected sensors should carry signal (i.e., there should be an SME), as timing analysis on sensors without an effect just leads to a flat curve. (2) Selection should be specific for each frequency band because gamma and theta effects appear at different locations. (3) Gamma and theta effects likely differ in spatial extent; therefore, selecting a fixed number of sensors helps selecting spatially limited “hotspots.” (4) Thresholding (i.e., setting negative/positive values to zero) simplifies calculating the area under the curve and ensures that the analysis is not primarily driven by later/earlier effects in the opposing direction. S9 Fig shows that the results of the analysis remain stable when using more-liberal selection and thresholding criteria (i.e., average across all sensors, and no thresholding of SME curves). This area-under-the-curve procedure returns a time-resolved curve illustrating for each time point the percentage of the SME curve covered. To assess statistical differences in area-under-the-curve measure of theta and gamma SMEs, again a cluster permutation approach was utilized, shuffling the condition (theta/gamma) 1,000 times across subjects and clustering significant t-values along the time dimension.

## Supporting information

S1 Fig (A) Cluster statistics results corresponding to Fig 2. t-Sum plots show significant clusters exhibiting material-specific changes in lower (2.30 Hz) and higher frequencies (40–100 Hz), respectively. (B) Relative change to baseline in MEG (−600- to −100-ms prestimulus) separately for the “word” cluster (see Fig 2B, red frontal cluster) and (C) in the “face” cluster (see Fig 2B, blue posterior cluster). Note that relative increases and decreases largely match the selected time-frequency bands of interest and that the general pattern of differences is qualitatively similar to the reported results contrasting z-transformed data. MEG, magnetoencephalography.
(TIF)

S2 Fig (A and C) The overlap of MEG and iEEG material effects is visualized by plotting for each category of iEEG electrodes (2, “w < f”: light blue, “w > f”: orange, or no significant difference: gray); the percentage of electrodes located in areas exhibit nominally positive or negative differences in MEG (w < f: red, w > f: blue). This analysis is a replication of the analysis in Fig 2 using more-liberal criteria for categorizing negative/positive effects in MEG. The numbers plotted on top of the bars denote the absolute numbers of electrodes in each source area, respectively. χ^2^ Test of independence shows that distribution of iEEG effects and MEG differences is not independent (alpha/beta: χ^2^_2_ = 38.75, *p* < .0001, gamma χ^2^_2_ = 7.14, *p* = .028). (B and D) Scatterplots of differences of word–face alpha/beta or gamma power, respectively, in iEEG electrodes and the corresponding MEG source sensor. Both gamma power and alpha/beta power changes correlated significantly between iEEG electrodes and matching MEG source sensors. iEEG, intracranial electroencephalography; MEG, magnetoencephalography.
(TIF)

S3 Fig Results of a spatial cluster permutation analysis clustering across electrodes with a maximal distance of 2 cm. Orange highlights electrodes belonging to a significant positive cluster; blue highlights clusters belonging to a significant negative cluster. Note that the overall pattern of results is similar to reported uncorrected electrodes plots in Figs 2 and 4 and to permutation statistics based on significant electrodes numbers (Fig 4G).
(TIF)

S4 Fig Average mean and SD across all subjects and sensors in MEG used for z-transformation. Note that the average mean subtracted from the data when z-transforming the data already subtracts part of the frequency tilt by subtracting the average signal. MEG, magnetoencephalography.
(TIF)

S5 Fig Average power spectra of all conditions in SME ROIs (see Fig 4). Black and red dots mark uncorrected and FDR-corrected significant SMEs, respectively. Note that the difference in spacing of dots in higher and lower frequencies results from the difference in frequency resolution. The significance test here is directly depending on the reported results in Fig 4. Significance in this plot is solely highlighted to illustrate the frequency specificity of reported SMEs, i.e., that low-frequency negative SMEs do not necessarily coappear with positive high-frequency SMEs. FDR, false discovery rate; ROI, region of interest; SME, subsequent memory effect.
(TIF)

S6 Fig Results of the spectral tilt fit in MEG data in SME ROIs source cluster (see Fig 4). Bar plots show average fitted slopes for each condition and fit; dots mark individual subjects. Asterisks highlight significant effects. Note that task-related differences in slopes are varying with fitted frequency band. Raw power plots show the raw power spectrum in log–log space with fitted frequency tilts spanning the respectively fitted frequency bands. Residual power spectrum plots show power spectra after tilt correction; crosses highlight detected peaks in the spectrum. Black and red dots mark uncorrected or FDR-corrected significant SMEs, respectively. Note here that SMEs mostly remain stable after tilt correction, except for gamma effects, which in MEG vanish when correcting tilt. FDR, false discovery rate; MEG, magnetoencephalography; ROI, region of interest; SME, subsequent memory effect.
(TIF)

S7 Fig Results of the spectral tilt fit in iEEG data in SME ROI electrodes (see Fig 4). Bar plots show average fitted slopes for each condition and fit; dots mark single electrodes. Asterisks highlight significant effects. Note that task-related differences in slopes are varying with fitted frequency band. Raw power plots show the raw power spectrum in log–log space with fitted frequency tilts spanning the respective frequency bands used for fitting. Residual power spectrum plots show power spectra after tilt correction; crosses highlight detected peaks in the spectrum. Black and red dots mark uncorrected or FDR-corrected significant SMEs, respectively. Note here that SMEs mostly remain stable after tilt correction. FDR, false discovery rate; iEEG, intracranial electroencephalography; ROI, region of interest; SME, subsequent memory effect.
(TIF)

S8 Fig Simulation approach using Gaussian distributed curves with substantial differences in SDs demonstrating that the area-under-the-curve method is sensitive to latency difference also when contrasting signals with differences in smoothness. (TIF)

S9 Fig AUC latency analysis replicating the analysis in Fig 5H using different thresholding for area calculation and varying sensor definitions. (A) AUC for SME curves based on subject-specific averages across all source sensors and using no threshold for area calculation. (B) AUC for SME curves based on subject-specific averages across all source sensors and using a threshold for area calculation (theta SME area < 0, theta SME area > 0). (C) AUC for SME curves based on subject-specific averages across individual 5% sensor with highest average effects (200–1,500 ms) and using no threshold for area calculation. The average area under the SME curve reached at each time point in these frequency- and subject-specific ROIs is plotted for gamma and theta SMEs; the gray area highlights the significant cluster (random cluster permutation) in which gamma SMEs significantly precede theta SMEs. Plots below show the average SME curves. Note that the latency difference remains significant irrespective of used thresholding or sensor selection. AUC, area under the curve; ROI, region of interest; SME, subsequent memory effect.
(TIF)

S1 Text Supplementary methods: Spatial cluster permutations statistics iEEG. iEEG, intracranial electroencephalography.
(DOCX)

S2 Text Supplementary methods: Spectral tilt fit. (DOCX)

## Abbreviations

EEG: electroencephalography
ERP: event-related potential
FEA: fixed-effects analysis
fMRI: functional MRI
iEEG: intracranial EEG
MEG: magnetoencephalography
MTL: medial temporal lobe
ROC: receiver operating characteristic
ROI: region of interest
SME: subsequent memory effect
