# Supplementary figures and images for: Spectral fingerprints or spectral tilt? Evidence for distinct oscillatory signatures of memory formation
Source: PLoS Biol. 2019 Jul 29;17(7):e3000403. doi: 10.1371/journal.pbio.3000403 (PMC6687190; doi:10.1371/journal.pbio.3000403)

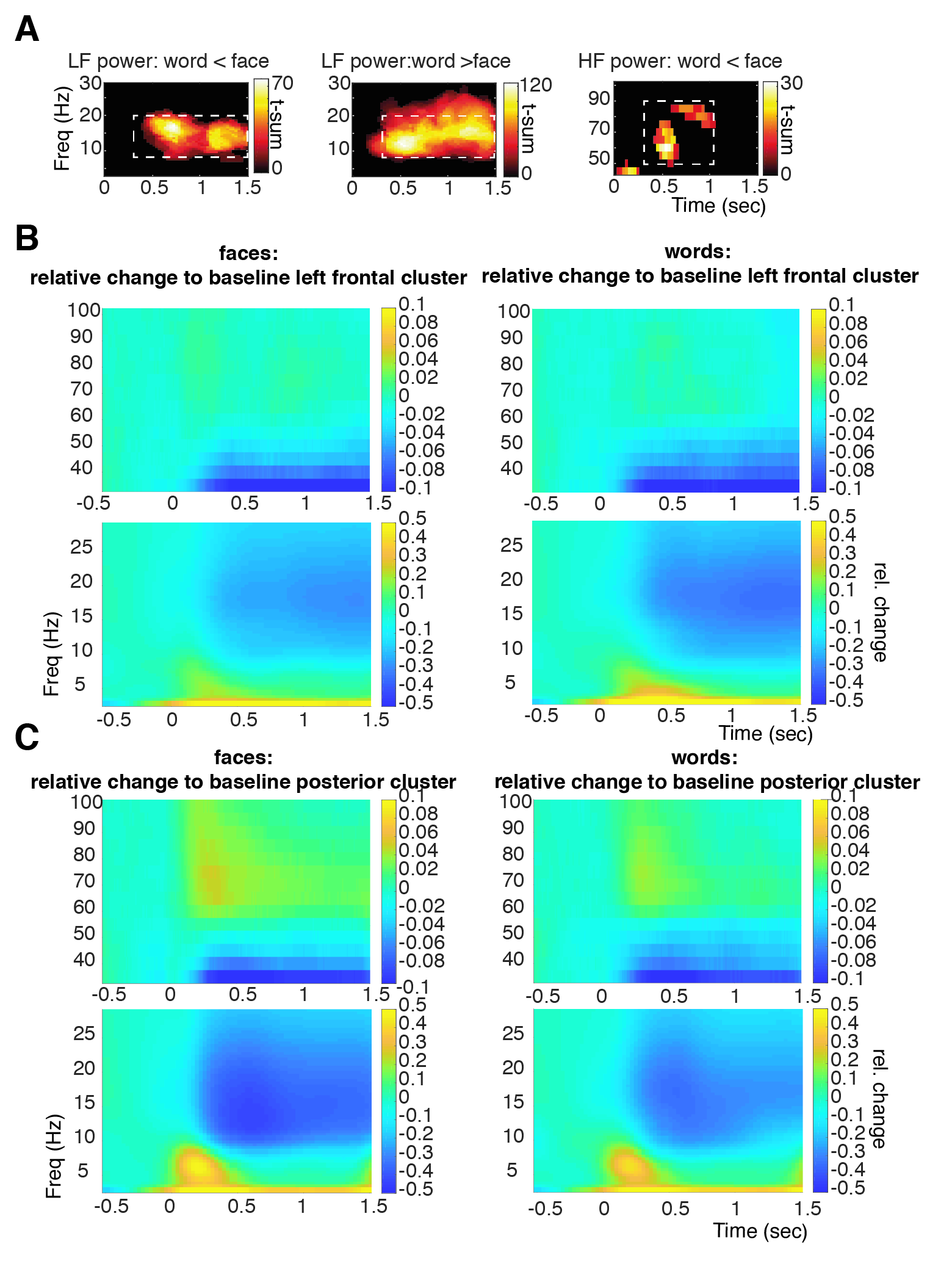

Supplement: S1 Fig — (A) Cluster statistics results corresponding to Fig 2. t-Sum plots show significant clusters exhibiting material-specific changes in lower (2.30 Hz) and higher frequencies (40–100 Hz), respectively. (B) Relative change to baseline in MEG (−600- to −100-ms prestimulus) separately for the “word” cluster (see Fig 2B, red frontal cluster) and (C) in the “face” cluster (see Fig 2B, blue posterior cluster). Note that relative increases and decreases largely match the selected time-frequency bands of interest and that the general pattern of differences is qualitatively similar to the reported results contrasting z-transformed data. MEG, magnetoencephalography. (TIF) [file pbio.3000403.s001.tif]

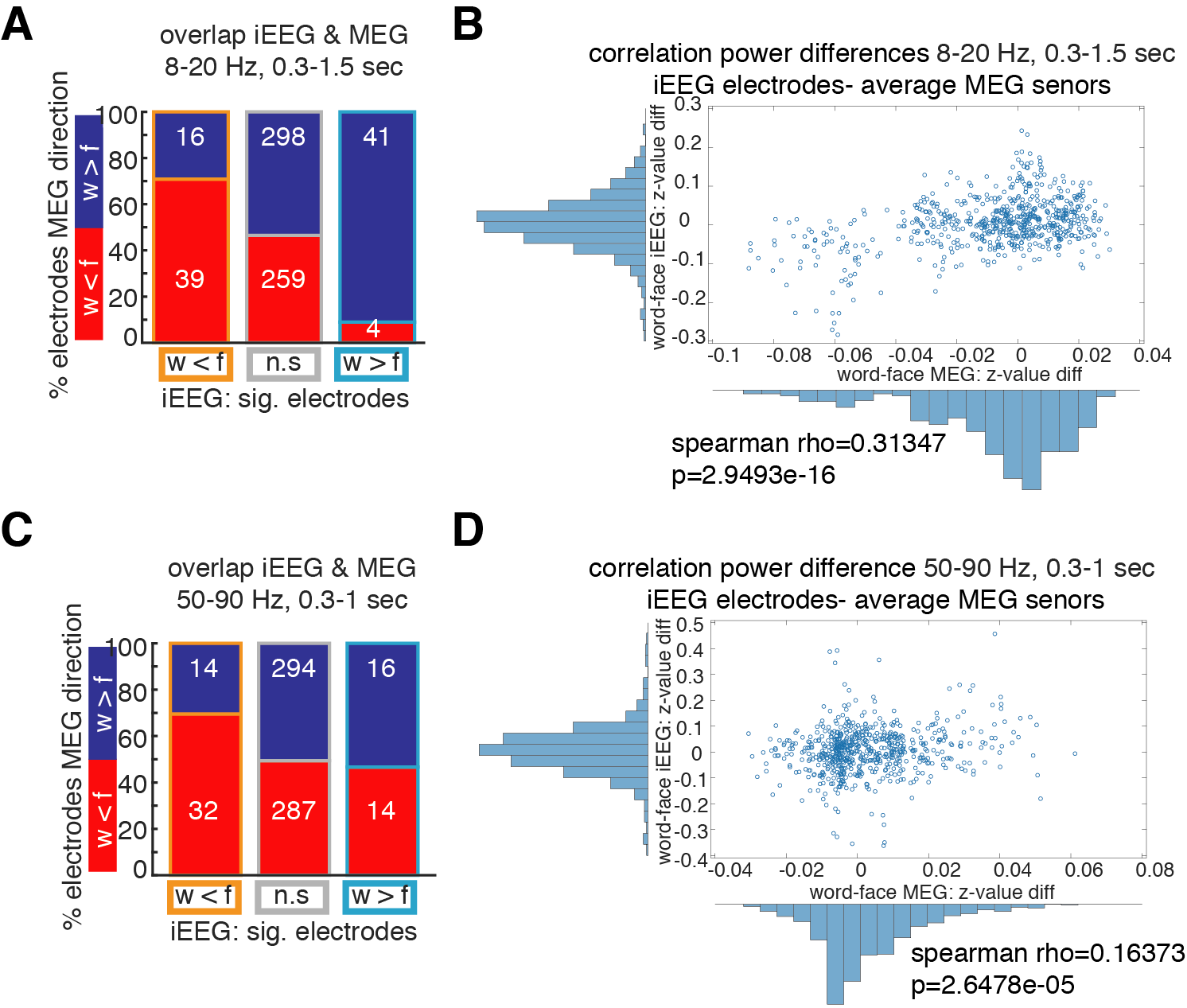

Supplement: S2 Fig — (A and C) The overlap of MEG and iEEG material effects is visualized by plotting for each category of iEEG electrodes (2, “w < f”: light blue, “w > f”: orange, or no significant difference: gray); the percentage of electrodes located in areas exhibit nominally positive or negative differences in MEG (w < f: red, w > f: blue). This analysis is a replication of the analysis in Fig 2 using more-liberal criteria for categorizing negative/positive effects in MEG. The numbers plotted on top of the bars denote the absolute numbers of electrodes in each source area, respectively. χ2 Test of independence shows that distribution of iEEG effects and MEG differences is not independent (alpha/beta: χ22 = 38.75, p < .0001, gamma χ22 = 7.14, p = .028). (B and D) Scatterplots of differences of word–face alpha/beta or gamma power, respectively, in iEEG electrodes and the corresponding MEG source sensor. Both gamma power and alpha/beta power changes correlated significantly between iEEG electrodes and matching MEG source sensors. iEEG, intracranial electroencephalography; MEG, magnetoencephalography. (TIF) [file pbio.3000403.s002.tif]

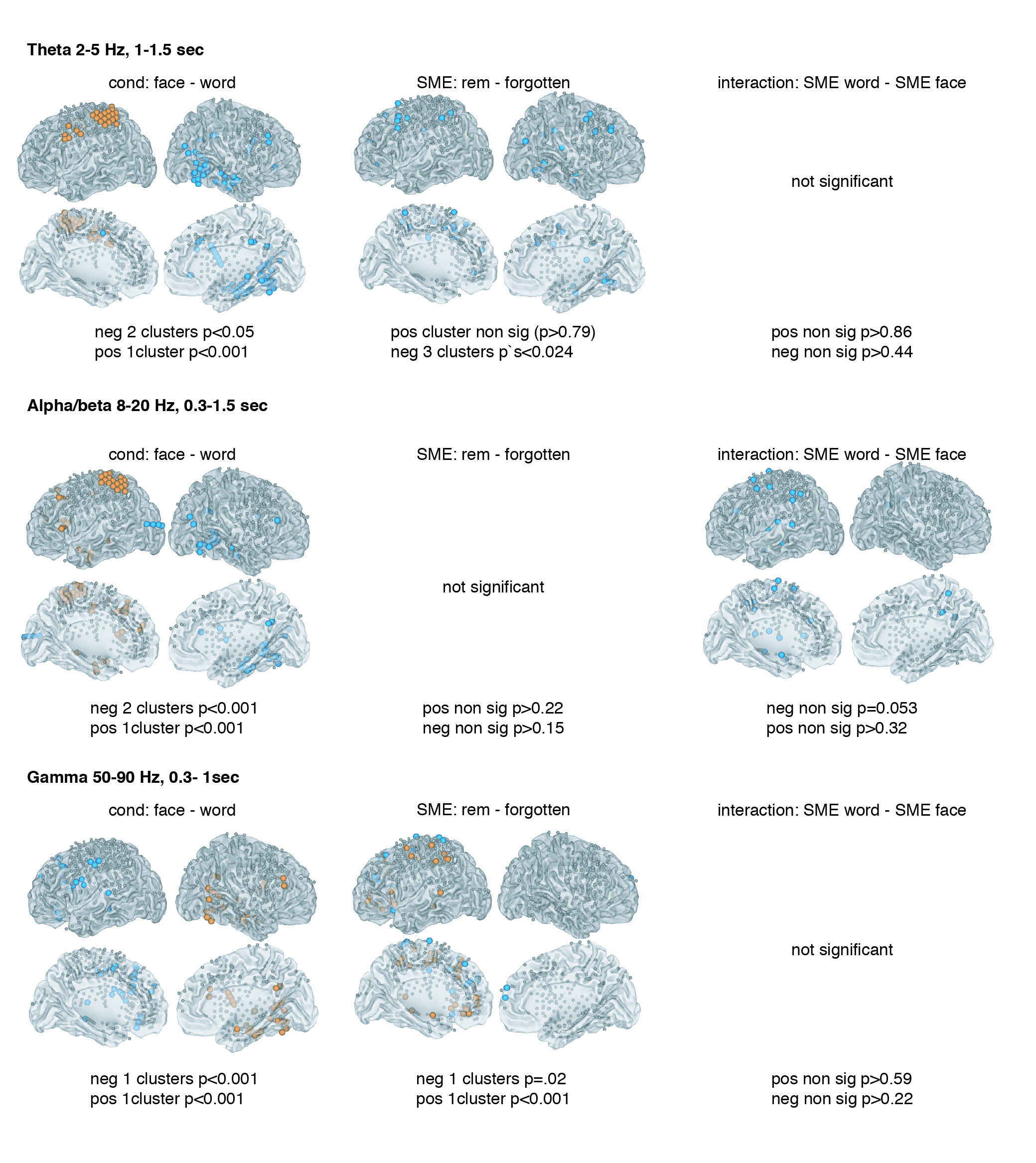

Supplement: S3 Fig — Orange highlights electrodes belonging to a significant positive cluster; blue highlights clusters belonging to a significant negative cluster. Note that the overall pattern of results is similar to reported uncorrected electrodes plots in Figs 2 and 4 and to permutation statistics based on significant electrodes numbers (Fig 4G). (TIF) [file pbio.3000403.s003.tif]

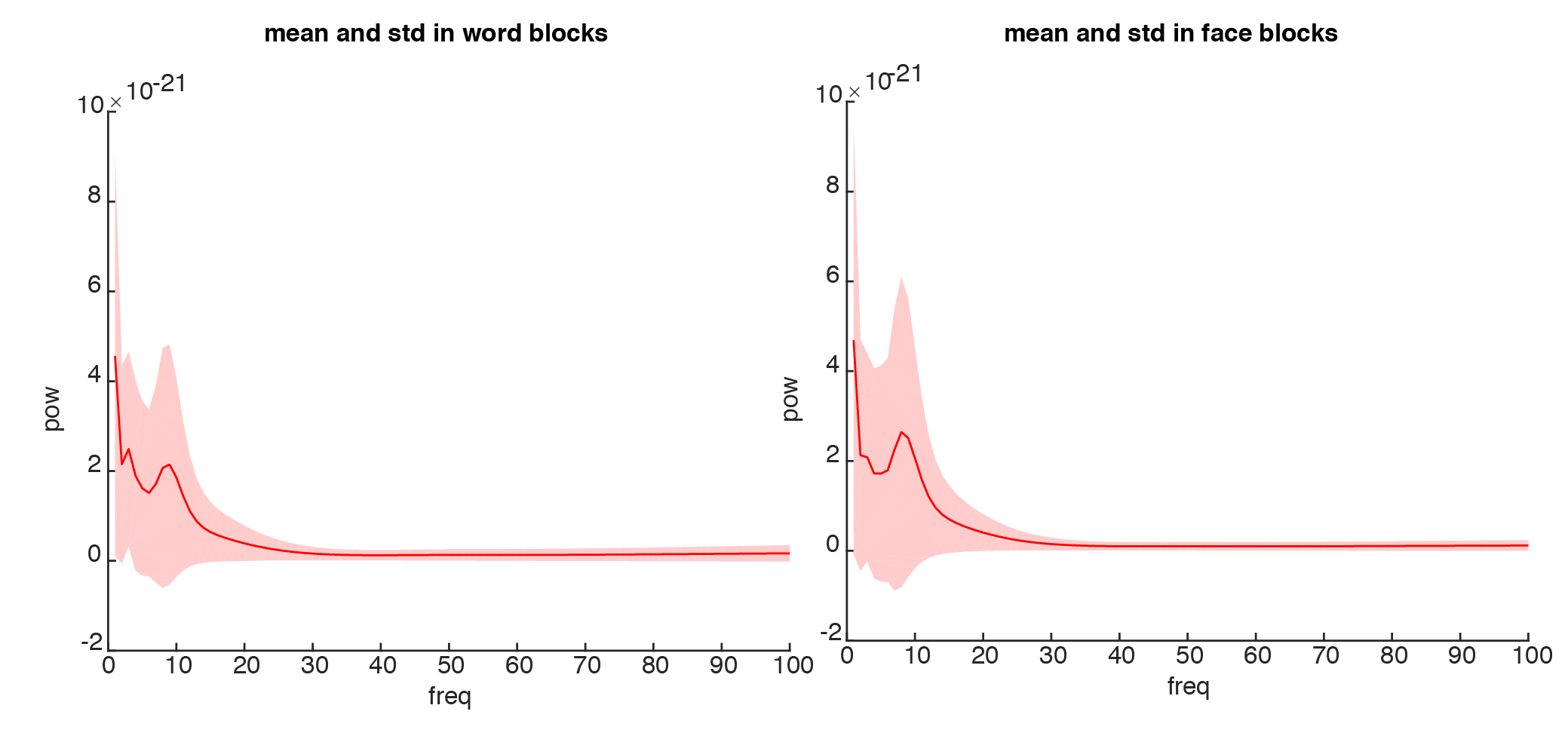

Supplement: S4 Fig — Note that the average mean subtracted from the data when z-transforming the data already subtracts part of the frequency tilt by subtracting the average signal. MEG, magnetoencephalography. (TIF) [file pbio.3000403.s004.tif]

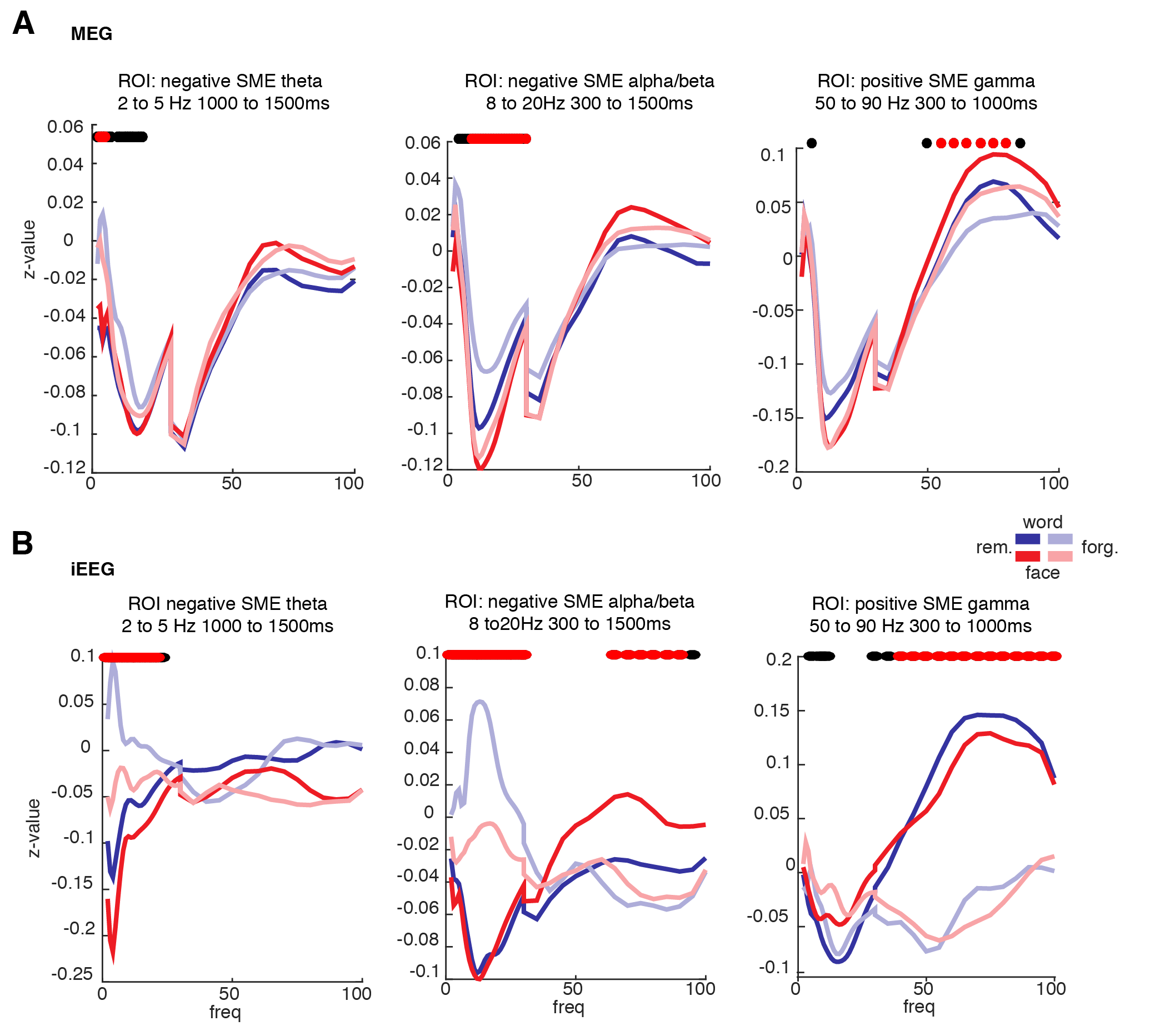

Supplement: S5 Fig — Black and red dots mark uncorrected and FDR-corrected significant SMEs, respectively. Note that the difference in spacing of dots in higher and lower frequencies results from the difference in frequency resolution. The significance test here is directly depending on the reported results in Fig 4. Significance in this plot is solely highlighted to illustrate the frequency specificity of reported SMEs, i.e., that low-frequency negative SMEs do not necessarily coappear with positive high-frequency SMEs. FDR, false discovery rate; ROI, region of interest; SME, subsequent memory effect. (TIF) [file pbio.3000403.s005.tif]

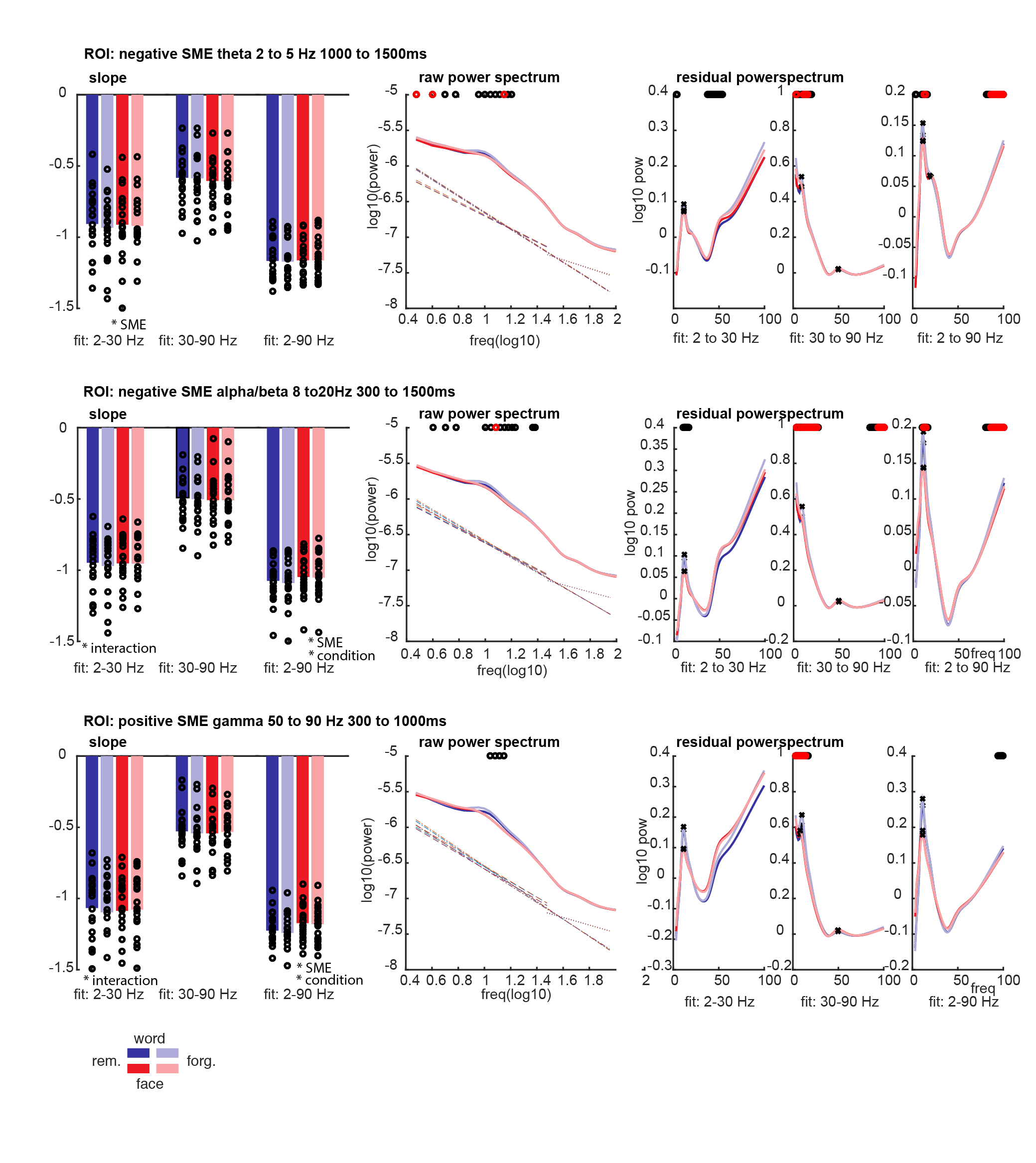

Supplement: S6 Fig — Bar plots show average fitted slopes for each condition and fit; dots mark individual subjects. Asterisks highlight significant effects. Note that task-related differences in slopes are varying with fitted frequency band. Raw power plots show the raw power spectrum in log–log space with fitted frequency tilts spanning the respectively fitted frequency bands. Residual power spectrum plots show power spectra after tilt correction; crosses highlight detected peaks in the spectrum. Black and red dots mark uncorrected or FDR-corrected significant SMEs, respectively. Note here that SMEs mostly remain stable after tilt correction, except for gamma effects, which in MEG vanish when correcting tilt. FDR, false discovery rate; MEG, magnetoencephalography; ROI, region of interest; SME, subsequent memory effect. (TIF) [file pbio.3000403.s006.tif]

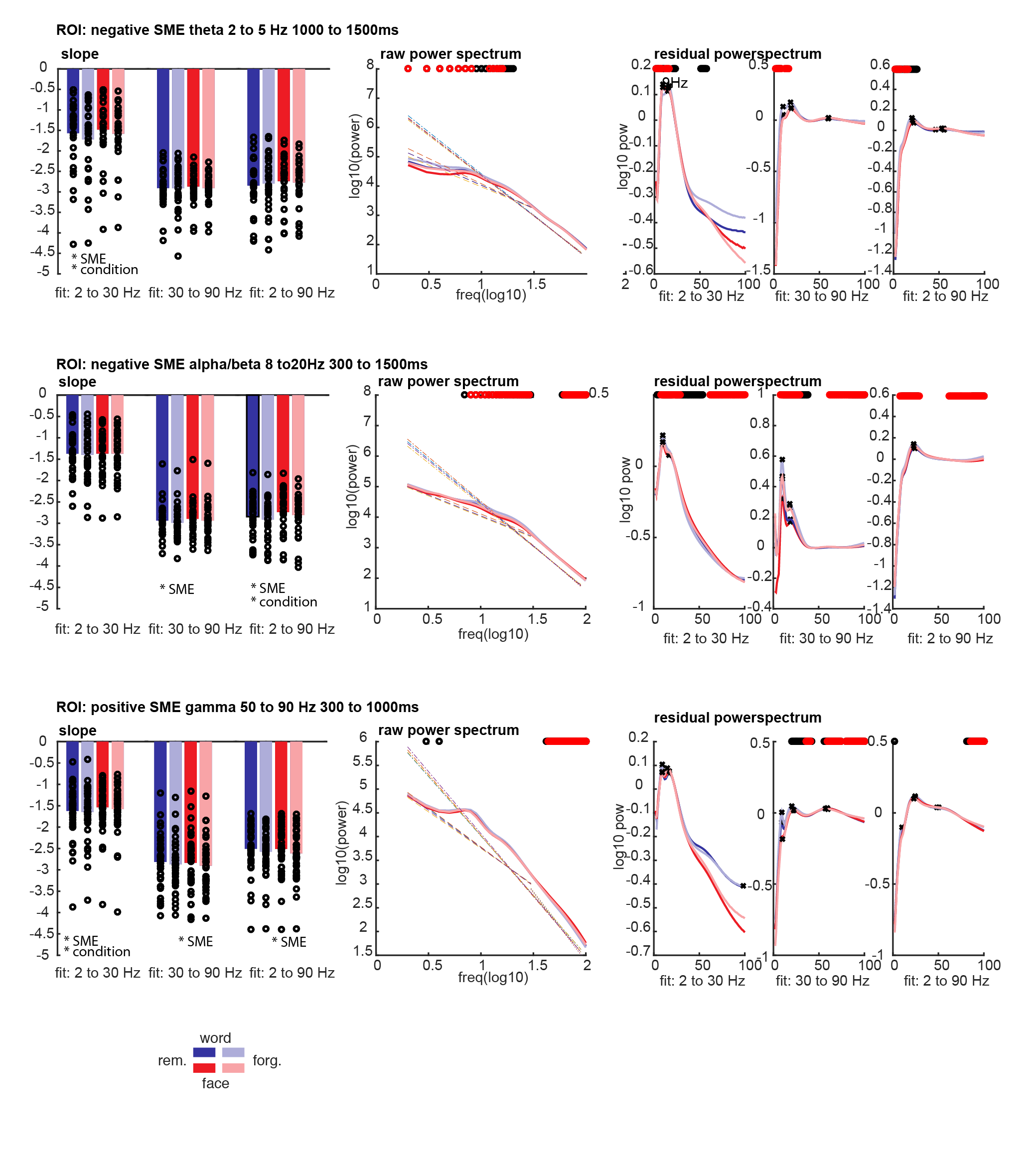

Supplement: S7 Fig — Bar plots show average fitted slopes for each condition and fit; dots mark single electrodes. Asterisks highlight significant effects. Note that task-related differences in slopes are varying with fitted frequency band. Raw power plots show the raw power spectrum in log–log space with fitted frequency tilts spanning the respective frequency bands used for fitting. Residual power spectrum plots show power spectra after tilt correction; crosses highlight detected peaks in the spectrum. Black and red dots mark uncorrected or FDR-corrected significant SMEs, respectively. Note here that SMEs mostly remain stable after tilt correction. FDR, false discovery rate; iEEG, intracranial electroencephalography; ROI, region of interest; SME, subsequent memory effect. (TIF) [file pbio.3000403.s007.tif]

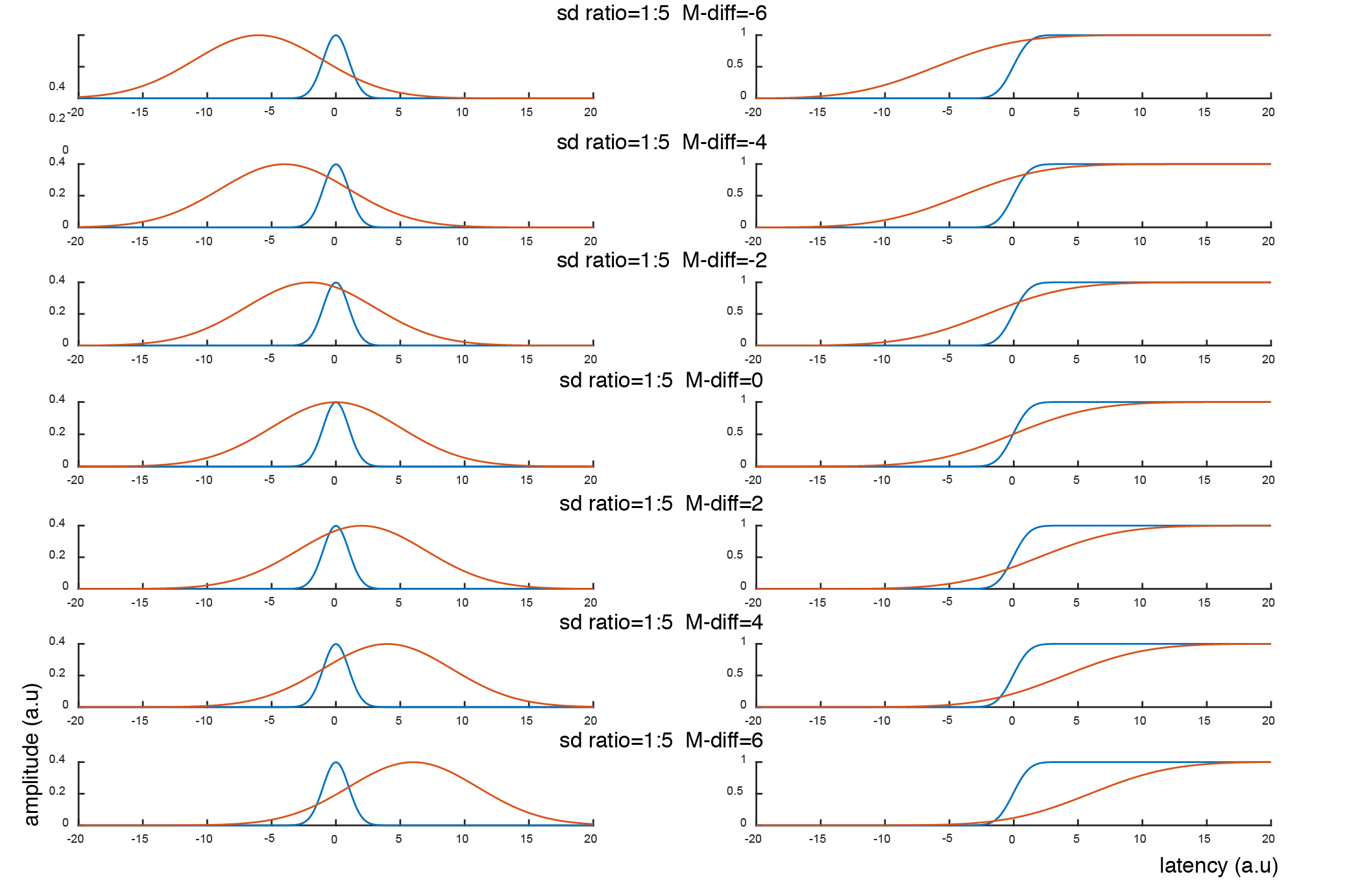

Supplement: S8 Fig — (TIF) [file pbio.3000403.s008.tif]

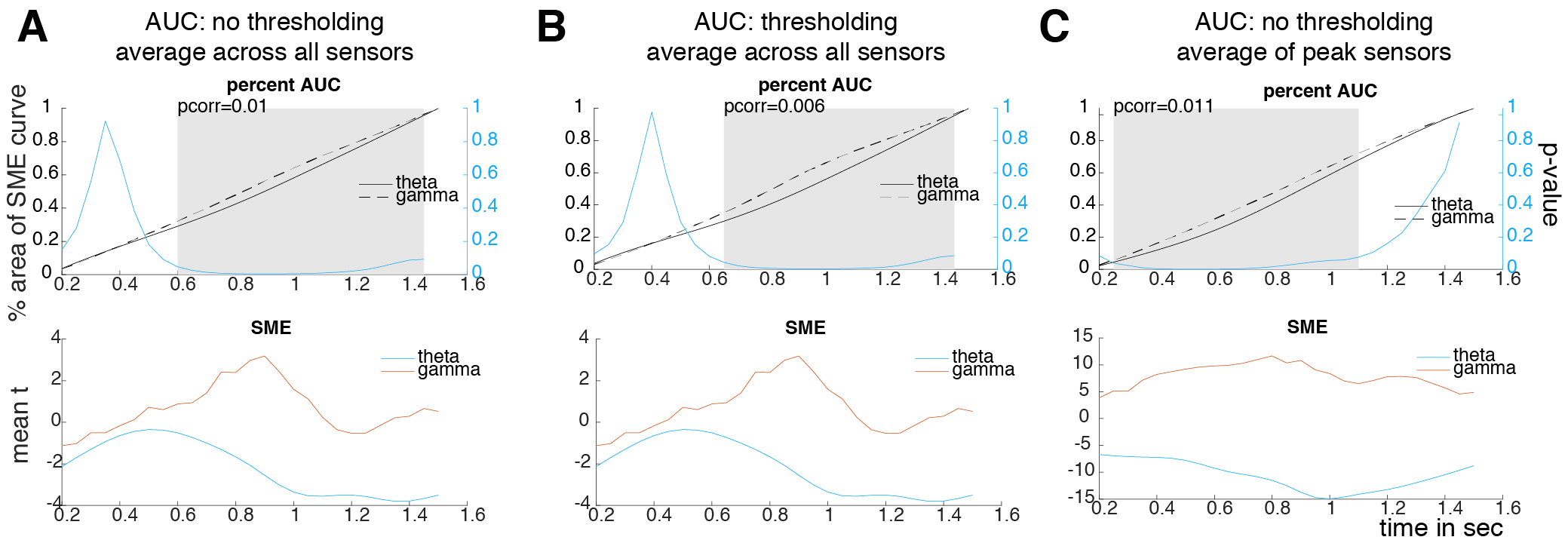

Supplement: S9 Fig — (A) AUC for SME curves based on subject-specific averages across all source sensors and using no threshold for area calculation. (B) AUC for SME curves based on subject-specific averages across all source sensors and using a threshold for area calculation (theta SME area < 0, theta SME area > 0). (C) AUC for SME curves based on subject-specific averages across individual 5% sensor with highest average effects (200–1,500 ms) and using no threshold for area calculation. The average area under the SME curve reached at each time point in these frequency- and subject-specific ROIs is plotted for gamma and theta SMEs; the gray area highlights the significant cluster (random cluster permutation) in which gamma SMEs significantly precede theta SMEs. Plots below show the average SME curves. Note that the latency difference remains significant irrespective of used thresholding or sensor selection. AUC, area under the curve; ROI, region of interest; SME, subsequent memory effect. (TIF) [file pbio.3000403.s009.tif]
