# Supplementary material for: Spectral fingerprints or spectral tilt? Evidence for distinct oscillatory signatures of memory formation
Source: PLoS Biol. 2019 Jul 29;17(7):e3000403. doi: 10.1371/journal.pbio.3000403 (PMC6687190; doi:10.1371/journal.pbio.3000403)
Supplement: S1 Text — iEEG, intracranial electroencephalography. (DOCX) [file pbio.3000403.s010.docx]

**Supplementary methods**

**Spatial cluster permutations statistics iEEG**

An additional spatial cluster permutation was carried out on iEEG data to further test the corroborate the reported results. Similar to the reported permutation statistics this analysis involved two calculation steps. In a step an ANOVA (material x memory) was calculated in every electrode, then spatial coherent clusters of significant positive/negative main effects or interaction effects were identified. Electrodes were defined as spatially neighboring if a maximal 2cm apart. F-Values of identified clusters were summed up. Significance was calculated by estimating the random distribution of F-sums by repeating the procedure 10000 times after randomly shuffling trial labels in each electrode (word-face, remembered-forgotten).

This clustering approach in iEEG data comes with serious limitations, which is why a different statistical approach for the iEEG data is reported in the paper. An important difference between MEG and iEEG is that MEG offers the exact same spatial coverage in every subject, which allows to cluster group based (i.e. random effects analysis) t-contrasts that uniformly cover every location in the brain. This is not the case for iEEG data where electrode placement is different from patient to patient (electrode position is purely dependent on the clinical needs of the patient). The here in the supplemental Figure 3 employed fixed-effects type cluster permutation analysis, must be interpreted with caution as neighboring electrodes are likely to belong to one patient, which makes this approach sensitive to outliers.
