# Supplementary material for: Spectral fingerprints or spectral tilt? Evidence for distinct oscillatory signatures of memory formation
Source: PLoS Biol. 2019 Jul 29;17(7):e3000403. doi: 10.1371/journal.pbio.3000403 (PMC6687190; doi:10.1371/journal.pbio.3000403)
Supplement: S2 Text — (DOCX) [file pbio.3000403.s011.docx]

**Spectral tilt fit**

To explore potential condition related changes in frequency tilt, we conducted an additional analysis using 1/f fitting. Based on prior published work a robust regression approach was used (robustfit.m Matlab function, see [1-3]. Slope and offset were fitted on condition specific, time resolved, in subject trial averaged raw power spectra (2-90 Hz, 5 cycle wavelets) in every sensor (MEG) or electrode (iEEG). This approach was used as single trial fitting of slopes necessary for reproducing iEEG stats yielded very unstable results. The sensor/subject specific slopes and offsets were then subtracted from the raw power spectra to yield a tilt corrected estimate of the power spectra.

As prior work used fits based on a wide variance of frequency bands (e.g. [1-4]) we repeated the fitting using three different frequency ranges (2-30Hz, 30-90Hz and 2-90Hz).

To explore the contribution of tilt shift to the reported effects, an ANOVA (material x memory) was carried out on the average slopes in every SME ROI (theta, alpha/beta, gamma source cluster/electrodes as plotted in Fig 4, see ANOVA results in supplemental Figure 6 & 7). Additionally, to check for stability of reported SMEs in tilt correct residual frequency spectra, SMEs were calculated for each frequency and fdr corrected across the number of frequency bands (see red dots in supplemental Fig 6 & 7).

**References**

1. Gao R, Peterson EJ, Voytek B. Inferring synaptic excitation/inhibition balance from field potentials. NeuroImage. 2017;158: 70–78. doi:10.1016/j.neuroimage.2017.06.078
2. Sheehan TC, ekumar V, Inati SK, Zaghloul KA. Signal Complexity of Human Intracranial EEG Tracks Successful Associative-Memory Formation across Individuals. The Journal of neuroscience : the official journal of the Society for Neuroscience. 2018;38: 1744–1755. doi:10.1523/JNEUROSCI.2389-17.2017
3. Manning JR, Jacobs J, Fried I, Kahana MJ. Broadband shifts in local field potential power spectra are correlated with single-neuron spiking in humans. The Journal of neuroscience : the official journal of the Society for Neuroscience. 2009;29: 13613–20. doi:10.1523/JNEUROSCI.2041-09.2009
4. Miller KJ, Sorensen LB, Ojemann JG, den Nijs M. Power-law scaling in the brain surface electric potential. PLoS computational biology. 2009;5: e1000609. doi:10.1371/journal.pcbi.1000609
